# Comparing the associations of eight surrogate markers of insulin resistance with rapid decline in kidney function and the incidence of CKD in Chinese middle-aged and elderly non-diabetic population

**DOI:** 10.1080/0886022X.2026.2633853

**Published:** 2026-04-07

**Authors:** Wei-Zhen Tang, Zhi-Yong Xiang, Qin-Yu Cai, Hong-Yu Xu, Zhi-Jian Zhou, Ting-He Sheng, Xu Zhang, Jun Ding, Tai-Hang Liu, Fei Han, Peng Guo

**Affiliations:** a The Third Affiliated Hospital of Chongqing Medical University, Chongqing, P. R. China; b Department of Bioinformatics, School of Basic Medical Sciences, Chongqing Medical University, Chongqing, P. R. China; c Department of Health Toxicology, School of Public Health, Chongqing Medical University, Chongqing, P. R. China

**Keywords:** Insulin resistance, AIP, CKD, rapid decline in kidney function, non-diabetic population

## Abstract

Estimated glucose disposal rate (eGDR), lipid accumulation product, Chinese visceral adiposity index, triglyceride-glucose, TyG-body mass index, TyG-waist circumference (TyG-WC), metabolic score for insulin resistance (METS-IR), and atherogenic index of plasma (AIP) are considered surrogate markers of insulin resistance (IR). However, there is currently a lack of comparative studies on the ability of different surrogate markers of insulin resistance to predict rapid decline in kidney function and the occurrence of chronic kidney disease (CKD) in non-diabetic populations. This study is based on data from the China Health and Retirement Longitudinal Study (CHARLS). Multivariable logistic regression models and trend regression analyses were used to examine the relationships between eight surrogate markers of insulin resistance and rapid decline in kidney function as well as the risk of CKD. Finally, the area under the curve of the surrogate markers of insulin resistance was calculated using receiver operating characteristic analysis. Each one SD increase in AIP was significantly associated with a reduced risk of rapid decline in kidney function and CKD, with adjusted ORs of 1.391 and 1.560, respectively. Dose-response analysis revealed nonlinear associations between TyG-WC and METS-IR with the risk of rapid decline in kidney function, and between eGDR and TyG-WC with CKD (P nonlinearity ≥ 0.05). ROC analysis indicated that AIP had significantly higher AUC values for predicting the risk of rapid decline in kidney function and CKD in the non-diabetic population, with AUCs of 0.676 and 0.608, respectively. AIP demonstrates strong potential in predicting the risk of rapid decline in kidney function and CKD in the Chinese middle-aged and elderly non-diabetic population, and this predictive ability is not influenced by sex or age.

## Introduction

CKD has become a major global public health issue over the past few decades. Since 1990, both the global incidence and mortality rates of CKD have shown a significant upward trend. Specifically, the global prevalence of CKD across all age groups has increased by 29.3%, and the corresponding global mortality rate has risen by 41.5% [1]. By 2040, CKD is projected to become the fifth leading cause of death worldwide. In countries with longer life expectancies, CKD is expected to rank among the top two causes of death by the end of this century [2]. The prevalence of CKD in China is approximately 10.8%, with an estimated 119.5 million individuals affected by the condition [3]. Kidney failure, the most severe stage of CKD, necessitates costly dialysis or kidney transplantation, imposing significant medical, economic, and social burdens on patients, families, and healthcare systems [4,5]. Therefore, identifying modifiable risk factors and reducing the burden of CKD and its related complications through early detection and primary prevention is of paramount importance.

Early detection and intervention are effective strategies to prevent the progression of kidney dysfunction to kidney failure. However, there is currently a lack of accurate and convenient indicators to predict rapid decline in kidney function. Various factors have been shown to contribute to kidney function decline, including glomerulonephritis, diabetes, hypertension, and nephrotoxic drugs. Since 2011, diabetes has replaced glomerulonephritis as the leading cause of CKD [6]. Among these, IR has been recognized as a key factor in the prevalence of type 2 diabetes and has been closely associated with the onset and progression of kidney disease. Insulin resistance impairs kidney function through various mechanisms, including reducing bioactive nitric oxide, affecting coagulation, activating the renin-angiotensin-aldosterone system, triggering oxidative stress, and initiating inflammatory responses, thereby impacting podocyte survival, glomerular filtration, renal tubular function, and leading to renal interstitial expansion and fibrosis [7,8]. However, despite hyperinsulinemic-euglycemic clamp being considered the gold standard for evaluating insulin resistance [9], its complexity, invasiveness, and high cost make it unsuitable for clinical and epidemiological research. As a result, various surrogate markers of insulin resistance have gained increasing attention. These markers have been shown not only to be valuable in assessing insulin resistance but also to be closely related to the risk and prognosis of cardiovascular diseases [10–12]. Recent studies have suggested that these markers may also be associated with rapid decline in kidney function and CKD [13–15].

In previous studies, the Chinese visceral fat index (CVAI), lipid accumulation product (LAP), triglyceride-glucose (TyG) index, and metabolic score for insulin resistance (METS-IR) have been considered effective and reliable indicators for assessing insulin resistance [16,17]. Regarding kidney function assessment, Liu et al. found that CVAI was superior to other indices in predicting CKD occurrence in middle-aged and elderly individuals [14]. Yu et al. observed a significant association between LAP and the prevalence of CKD in US adults [18]. Furthermore, recent studies have shown that the TyG index is associated with kidney function decline [19,20]. Abdominal obesity has also been recognized as a risk factor for insulin resistance [21]. Previous studies have used BMI and waist circumference (WC) as measures of abdominal obesity, and by multiplying the TyG index by BMI and WC, its predictive significance was enhanced. This approach demonstrated that TyG-WC had the highest predictive value for rapid decline in kidney function in middle-aged and elderly individuals [14]. The atherogenic index of plasma (AIP), a simple marker for assessing atherosclerosis, includes both triglycerides (TG) and high-density lipoprotein cholesterol (HDL-C). Recent studies have shown that AIP is an effective indicator for predicting the risk of a decrease in estimated glomerular filtration rate (eGFR) [22]. Additionally, the estimated glucose disposal rate (eGDR) has recently become a focal point in the evaluation of insulin resistance. Although previous studies have demonstrated that eGDR is more comprehensive and stable in predicting all-cause and cardiovascular mortality, there has been limited research on its use in kidney function assessment [23,24].

Given that diabetes is a major risk factor for CKD, and diabetic patients tend to exhibit more comorbidities and higher cardiovascular risk, which indirectly affects kidney function [25,26], most studies have focused on populations with diabetes or directly examined the role of insulin resistance markers in predicting kidney function in the general population [14,27,28]. However, the utility of these markers in predicting rapid decline in kidney function and CKD risk remains controversial, as previous studies have not fully considered the impact of factors such as sex and age, nor have they adjusted for additional confounding factors that may influence the results, potentially exaggerating or confounding the role of insulin resistance. For the larger non-diabetic population, although previous studies have generally suggested that non-diabetic CKD patients often exhibit insulin resistance [29,30], there is currently a lack of research comparing the predictive value of more comprehensive surrogate markers of insulin resistance for rapid decline in kidney function and CKD risk in individuals with impaired glucose metabolism. This study aims to analyze data from the CHARLS to evaluate the associations between eight surrogate markers of insulin resistance and rapid decline in kidney function and CKD risk, as well as their predictive value for these outcomes. Through this study, we hope to identify effective screening and preventive measures, and management strategies (such as optimizing blood glucose control, lipid management, and blood pressure management), thereby reducing the risk of kidney disease.

## Methods

### Study design and population

The detailed study design and methodology of CHARLS have been previously reported [31]. Briefly, CHARLS is a nationally representative longitudinal study aimed at collecting a wide range of demographic, socioeconomic, lifestyle, and health-related data from the middle-aged and elderly population in China. The baseline survey was conducted from June 2011 to March 2012, enrolling 17,708 participants aged 45 and above from 450 villages across 28 provinces. A multi-stage, stratified, and probability-proportional-to-size sampling method was used. All participants underwent one-on-one interviews using structured questionnaires. Follow-up data collection occurred every two years via face-to-face interviews. Each follow-up included physical measurements, and blood samples were collected every two follow-up cycles (i.e. every 4 years).

**Figure 1.**
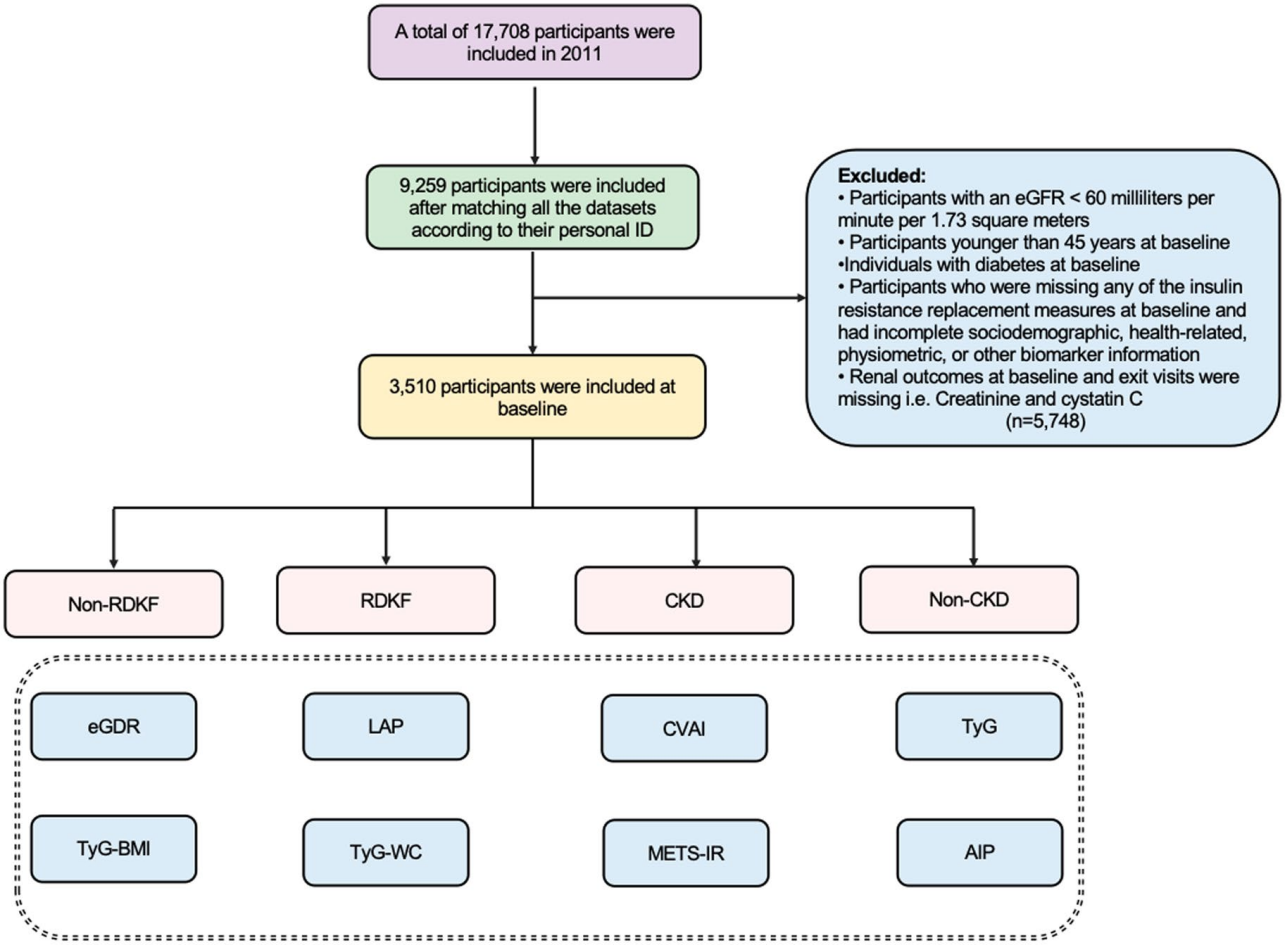
Flowchart of participant selection. RDKF: rapid decline in kidney function; CKD: chronic kidney disease; eGDR: estimated glucose disposal rate; LAP: lipid accumulation product; CVAI: Chinese Visceral Adiposity Index; TyG: triglyceride-glucose index; TyG-BMI: TyG-body mass index; TyG-WC: triglyceride-glucose-waist circumference; METS-IR: metabolic syndrome insulin resistance score; AIP: Atherogenic Index of Plasma.

We performed a prospective longitudinal analysis using data from three CHARLS waves in 2011, 2013, and 2015. To streamline the study sample, the following exclusion criteria were applied [1]: participants with kidney disease at baseline or with an eGFR <60 mL/min per 1.73 m^2^ at baseline [2]; participants younger than 45 years at baseline [3]; individuals with diabetes at baseline [4]; participants with missing data on any surrogate marker of insulin resistance, as well as incomplete data on sociodemographic, health-related, anthropometric, or other biomarkers [5]; participants with missing kidney outcomes, such as creatinine and cystatin C, at baseline or exit visits. After applying these exclusion criteria, 3,530 participants were included in the final analysis, with the exclusion process depicted in Figure 1. Figure 1 provides a diagrammatic description of the comprehensive selection process followed for participant recruitment.

The CHARLS project has received approval from the Biomedical Ethics Review Committee of Peking University (IRB00001052-11015). All participants provided written informed consent. Data and research materials supporting the findings of this study will be available on the CHARLS project website (http://charls.pku.edu.cn/).

### Data collection and measurements

During the baseline survey, interviewers collected data through questionnaires on sociodemographic factors (such as sex, age, education, marital status, household registration status, and retirement status), health-related behaviors (including smoking and alcohol consumption), and medical history (such as diabetes, hypertension, and heart disease). Educational attainment was categorized as no formal education, primary school, junior high school, and high school or above. Marital status was classified as married and other marital statuses (separated, divorced, widowed, and never married). Household registration status was categorized as rural and urban, while smoking and alcohol consumption status were also recorded. Anthropometric measurements, including height, weight, WC, systolic blood pressure (SBP), and diastolic blood pressure (DBP), were taken by trained professional staff.

### Laboratory assay

Trained personnel collected venous blood samples, which were transported at 4 °C to local laboratories. The blood samples were then stored at −20 °C before being shipped within two weeks to the China Centers for Disease Control and Prevention (CDC) in Beijing, where they were stored at −80 °C until analysis at the Capital Medical University laboratory. Serum glucose and lipids were measured using enzyme-linked colorimetric assays. Hemoglobin A1c (HbA1c) was determined using boronate affinity high-performance liquid chromatography. Uric acid was measured using an enhanced uric acid method. High-sensitivity C-reactive protein (hs-CRP) was measured using an immunoturbidimetric assay. Serum creatinine and cystatin C were measured using the Jaffe rate blank method and particle-enhanced turbidimetric immunoassay, respectively. Notably, in CHARLS, blood samples were collected during the 2011 and 2015 visits. Therefore, in our study, serum creatinine and cystatin C were assessed only at baseline (2011) and exit visits (2015).

### Calculation of eight surrogate markers of insulin resistance

eGDR, LAP, CVAI, TyG index, TyG-BMI, TyG-WC, METS-IR, and AIP were calculated using the following equations:

$$\text{BMI}={\text{weight}}_{\text{kg}}/{\text{height}}_{m}{}^{2}$$

$$\text{eGDR}=21.158-0.09\times {\text{WC}}_{\text{cm}}-3.407\times {\text{hypertension}}_{\left(\text{yes }=\text{ 1}/\text{no }=0\right)}-0.551\times {\text{HbA1c}}_{\%}.$$

$$\text{LAP }\left(\text{Male}\right)=\left[\left(\text{WC }\left(\text{cm}\right)-65\right)\times \text{TG }\left(\text{mmol}/L\right)\right]$$

$$\text{LAP }\left(\text{Female}\right)=\left[\left(\text{WC }\left(\text{cm}\right)-58\right)\times \text{TG }\left(\text{mmol}/L\right)\right]$$

$$\text{CVAI }\left(\text{male}\right)=-267.93+0.68\times {\text{age}}_{\text{years}}+0.03\times \text{BMI}+4.00\times {\text{WC}}_{\text{cm}}+22.00\times \text{ log}{\;}_{10}\;\left({\text{TG}}_{\text{mmol}/L}\right)-16.32\times {\text{HDL‐C}}_{\text{mmol}/L}.$$

$$\text{CVAI }\left(\text{female}\right)=-187.32+1.71\times {\text{age}}_{\text{years}}+4.23\times \text{BMI}+1.12\times {\text{WC}}_{\text{cm}}+39.76\times \text{ log}{\;}_{10}\;\left({\text{TG}}_{\text{mmol}/L}\right)-11.66\times {\text{HDL‐C}}_{\text{mmol}/L}.$$

$$\text{TyG index}=\text{ ln }\left({\text{TG}}_{\text{mg}/\text{dl}}\times {\text{FPG}}_{\text{mg}/\text{dl}}/2\right).$$

TyG‐BMI=TyG×BMI.

$$\text{TyG‐WC}=\text{TyG}\times \text{WC }\left(\text{cm}\right).$$

$$\text{METS‐IR}=\text{ ln }\left(2\times {\text{FPG}}_{\text{mg}/\text{dl}}+{\text{TG}}_{\text{mg}/\text{dl}}\right)\times \text{BMI}/\text{ ln }({\text{HDL‐C}}_{\text{mg}/\text{dl}}).$$

$$\text{AIP}=\text{ log }\left({\text{TG}}_{\text{mg}/\text{dl}}/{\text{HDL‐C}}_{\text{mg}/\text{dl}}\right).$$

### Renal function estimation

In this study, we primarily used the CKD-EPI Creatinine-Cystatin C equation developed by the Chronic Kidney Disease Epidemiology Collaboration (CKD-EPI) in 2021 [32] to assess kidney function. Although numerous previous studies have shown that eGFR based on the CKD-EPI Cystatin C equation can serve as a potential evaluation marker [33,34], recent findings indicate that the eGFR estimated by the CKD-EPI equation combining creatinine and cystatin C demonstrates higher accuracy when compared to the measured glomerular filtration rate (mGFR) [35]. In this study, serum creatinine was measured using the rate-blank compensated Jaffe method, while serum cystatin C was assessed using the particle-enhanced turbidimetric immunoassay. It is noteworthy that in the CHARLS, blood samples were collected in 2011 and 2015. Therefore, our study evaluated serum creatinine and cystatin C levels only at baseline (2011) and exit visits (2015).

### Definition

Diabetes was defined as self-reported physician diagnosis, use of antidiabetic medications, fasting blood glucose ≥126 mg/dL, or hemoglobin A1c ≥6.5% [36]. Hypertension was defined as self-reported physician diagnosis, use of antihypertensive medications, or an average systolic/diastolic blood pressure ≥140/90 mmHg [37].

The outcome variables of this study were rapid decline in kidney function and the incidence of CKD. The primary outcome was rapid decline in kidney function, defined as a yearly eGFRcr-cys decline of 5 mL/min per 1.73 m^2^ or more, based on prior studies using data from the CHARLS [38–41]. The yearly eGFRcr-cys decline was estimated as (baseline eGFRcr-cys − exit eGFRcr-cys)/follow-up time [42]. The secondary outcome was the occurrence of CKD, defined as a decline in eGFR to <60 mL/min per 1.73 m^2^ at the exit visit [14].

### Statistical analysis

Continuous variables are expressed as the median and IQR, and categorical variables are presented as frequencies and percentages. Comparisons between groups were made using independent samples *t*-tests, Mann-Whitney *U* tests, or chi-square tests. The correlations between the eight surrogate markers of IR and the risk of rapid decline in kidney function and CKD were assessed using logistic regression analysis. To facilitate direct comparison of ORs, the eight IR surrogate markers were converted into Z-scores. Three logistic regression models were used in this study. Model 1 was unadjusted for any variables. Model 2 adjusted for sex, age, smoking status, alcohol consumption, marital status, education level, household registration status, retirement status, BMI, and WC. Model 3 adjusted for sex, age, smoking status, alcohol consumption, marital status, education level, household registration status, retirement status, BMI, WC, total cholesterol (TC), low-density lipoprotein cholesterol (LDL-C), serum creatinine (Scr), cystatin C (Cys), uric acid (SUA), blood urea nitrogen (BUN), C-reactive protein (CRP), hypertension, and heart disease. For all IR surrogate markers, the variables already included in the equation were not adjusted for in the regression models. This was done to avoid potential multicollinearity, as including these variables separately could lead to highly correlated predictors, distorting the regression results and affecting the precision of the coefficient estimates. Multicollinearity between variables in each model was assessed using the variance inflation factor (VIF). The VIF values for all variables in each model were below 10, indicating no significant multicollinearity issues (Table S1). Furthermore, we explored the dose-response relationship between IR surrogate markers and the risk of rapid decline in kidney function and CKD using restricted cubic splines (RCS). The reference level for ORs was the median value of the IR surrogate markers. The baseline was set at *Y* = 1. To further examine the dose-response relationship between exposure to IR surrogate markers and the risk of rapid decline in kidney function and CKD, the distribution of the IR surrogate markers was divided into quartiles (Q1, Q2, Q3, Q4). Trend regression analysis was conducted, using the lowest quartile (Q1) as the reference group, to evaluate the changing trends in outcome risk from the Q1 group to the Q4 group. The predictive ability of these markers for rapid decline in kidney function and CKD risk was evaluated using receiver operating characteristic (ROC) curves. The area under curve (AUC), optimal cutoff values, sensitivity, specificity, and Youden’s index (sensitivity + specificity − 1) were calculated for each marker. We also used the DeLong test to assess differences in AUCs between the different IR surrogate markers. All P-values were two-tailed, with *p* < 0.05 considered statistically significant. All analyses were performed using SPSS 26.0 and R 4.4.2 software for statistical analysis.

## Results

### Baseline characteristics of study participants

A total of 3,530 participants were included in this study, comprising 1,657 men and 1,873 women. Table 1 presents the baseline clinical and biochemical characteristics, stratified by the presence or absence of future rapid decline in kidney function and CKD, as assessed by eGFRcr-cys (Table 1). The results show that among individuals with rapid decline in kidney function, 44.72% were male (72 individuals). This group exhibited significantly higher levels of SBP, BMI, WC, TC, TG, BUN, Scr, CRP, FPG, LAP, CVAI, TyG index, TyG-BMI, TyG-WC, METS-IR, and AIP, while levels of HDL-C, eGDR, Scr, and Cys were significantly lower (*p* < 0.05). In the CKD group, 45.35% were male (39 individuals). This group had significantly higher age, proportion of individuals with lower education, prevalence of hypertension, SBP, TG, SUA, BUN, CRP, Scr, Cys, LAP, TyG index, TyG-WC, and AIP levels (*p* < 0.05), while HDL-C and eGDR levels were significantly lower (*p* < 0.01).

**Table 1.** Baseline characteristics of study participants with or without rapid decline in kidney function and CKD, as calculated by eGFRcr-cys.

	Rapid decline in kidney function in follow-up			CKD in follow-up		
	Yes	No		Yes	No	
Characteristics	*n* (%)	*n* (%)	*p*	*n* (%)	*n* (%)	*p*
No.						
Sex			0.563			0.765
Female	89 (55.28)	1784 (52.95)		47 (54.65)	1826 (53.02)	
Male	72 (44.72)	1585 (47.05)		39 (45.35)	1618 (46.98)	
Age (years)			0.402			<0.001^*^
45–60	78 (48.45)	1746 (51.83)		12 (13.95)	1812 (52.61)	
≥60	83 (51.55)	1623 (48.18)		74 (86.05)	1632 (47.39)	
Smoking	58 (36.03)	1335 (39.63)	0.361	37 (43.02)	1356 (39.37)	0.494
Alcohol consumption	61 (37.89)	1324 (39.31)	0.718	29 (33.72)	1356 (39.38)	0.288
Marital status			0.319			0.076
Married/cohabitating	137 (85.09)	2956 (87.74)		70 (81.40)	3023 (87.78)	
Divorced/separated/ widowed/never married	24 (14.91)	413 (12.26)		16 (18.61)	421 (12.22)	
Education level			0.339			0.011^*^
Illiterate	89 (55.28)	1732 (51.44)		58 (67.44)	1763 (51.22)	
Primary school or below	41 (25.47)	776 (23.05)		18 (20.93)	799 (23.21)	
Middle school	23 (14.29)	606 (18.00)		6 (6.98)	623 (18.10)	
High school or above	8 (4.97)	253 (7.51)		4 (4.65)	257 (7.47)	
Residence			0.627			0.969
Rural	144 (89.44)	2969 (88.18)		76 (88.37)	3037 (88.23)	
Urban	17 (10.56)	398 (11.82)		10 (11.63)	405 (11.77)	
Retired	7 (4.38)	212 (6.42)	0.300			
Hypertension, n (%)	45 (28.13)	799 (23.83)	0.214	37 (43.02)	807 (23.55)	<0.001^*^
Heart diseases, n (%)	12 (7.50)	375 (11.21)	0.144	13 (15.12)	374 (10.94)	0.222
SBP (mmHg)	141.00 (123.00,156.00)	131.00 (118.00,147.00)	<0.001^*^	141.00 (128.00,156.00)	131.00 (118.00,147.00)	<0.001^*^
DBP (mmHg)	80.00 (71.00,87.00)	77.00 (69.00,86.00)	0.093	78.00 (70.00,86.00)	77.00 (69.00,86.00)	0.675
BMI (kg/m^2^)	23.57 (21.49,26.29)	22.83 (20.60,25.44)	0.002^*^	23.09 (21.05,26.00)	22.86 (20.65,25.47)	0.608
WC (cm)	87.20 (79.60,93.20)	84.00 (77.20,91.00)	0.004^*^	86.00 (77.80,94.00)	84.00 (77.30,91.00)	0.069
TC (mg/dl)	200.26 (173.20,224.62)	190.98 (167.78,214.56)	0.014^*^	190.98 (165.85,215.34)	191.37 (167.78,215.34)	0.757
TG (mg/dl)	150.45 (92.93,250.46)	102.66 (73.46,147.80)	<0.001^*^	121.25 (85.85,176.12)	103.55 [73.46,149.57)	0.003^*^
HDL-C (mg/dl)	42.91 (35.18,51.03)	50.26 (41.37,61.08)	<0.001^*^	45.62 (35.57,56.44)	49.87 (41.37,60.70)	0.002^*^
LDL-C (mg/dl)	114.05 (86.21,136.86)	114.43 (93.94,136.86)	0.192	117.14 (91.24,141.11)	114.43 (93.56,136.86)	0.713
SUA (mg/dl)	4.42 (3.46,5.26)	4.23 (3.54,5.04)	0.246	4.66 (4.24,5.47)	4.22 (3.52,5.04)	<0.001^*^
BUN (mg/dl)	14.01 (12.04,17.11)	15.15 (12.60,18.01)	0.015^*^	16.92 (14.03,19.27)	15.10 (12.52,17.93)	<0.001^*^
Scr (mg/L)	0.70 (0.60,0.83)	0.75 (0.64,0.86)	0.003^*^	0.85 (0.71,0.98)	0.73 (0.64,0.850	<0.001^*^
Cys (mg/L)	0.800 [0.660,0.920]	0.980 [0.870,1.100]	<0.001^*^	1.16 (1.01,1.28)	0.97 (0.86,1.09)	<0.001^*^
CRP (mg/L)	1.22 (0.59,2.41)	0.97 (0.55,1.99)	0.024^*^	1.53 (0.85,2.92)	0.97 (0.54,1.99)	<0.001^*^
FPG (mg/dl)	106.20 (95.76,119.52)	101.70 (94.14,110.52)	<0.001^*^	100.44 (93.42,115.92)	101.88 (94.32,110.88)	0.558
HbA1c (%)	5.20 (4.90,5.50)	5.10 (4.90,5.40)	0.299	5.20 (5.00,5.50)	5.10 (4.90,5.40)	0.191
eGDR	10.21 (7.99,11.04)	10.54 (8.92,11.33)	<0.001^*^	9.89 (6.85,11.16)	10.54 (8.98,11.32)	<0.001^*^
LAP	42.15 (21.35,77.94)	25.36 (13.64,44.69)	<0.001^*^	36.93 (14.83,65.07)	25.71 (13.75,45.59)	0.011^*^
CVAI	108.06 (73.48,139.24)	90.27 (64.63,120.38)	<0.001^*^	97.88 (70.00,131.41)	90.89 (64.80,121.26)	0.167
TyG	9.02 (8.44,9.61)	8.56 [8.20,8.98]	<0.001^*^	8.77 (8.34,9.13)	8.56 (8.21,8.99)	0.005^*^
TyG-BMI	215.69 (183.95,248.97)	195.58 (173.26,224.10)	<0.001^*^	201.54 (177.68,244.39)	201.54 (177.68,244.39)	0.177
TyG-WC	792.56 (675.14,870.90)	718.50 (645.14,801.66)	<0.001^*^	749.02 (649.49,858.74)	719.93 (646.27,805.51)	0.032^*^
METS-IR	38.03 [31.90,45.48]	33.57 [29.25,39.27]	<0.001^*^	35.23 (29.04,46.36)	33.67 (29.31,39.44)	0.092
AIP	0.53 (0.29,0.83)	0.31 [0.11,0.52)	<0.001^*^	0.47 (0.15,0.67)	0.31 (0.11,0.53)	<0.001^*^

*Abbreviation:* SBP: systolic blood pressure; DBP: diastolic blood pressure; BMI: body mass index; WC: waist circumference; TC: total cholesterol; TG: triglyceride; HDL-C: high-density lipoprotein cholesterol; LDL-C: low-density lipoprotein cholesterol; SUA: serum uric acid; BUN: blood urea nitrogen; Scr: serum creatinine; Cys: Cystatin C; CRP: C-reactive protein; FPG: fasting plasma glucose; HbA1c: glycosylated hemoglobin A1c; eGDR: estimated glucose disposal rate; LAP, Lipid Accumulation Product; CVAI: Chinese visceral adiposity index; TyG: triglyceride-glucose; TyG-BMI: TyG-body mass index; TyG-WC: TyG-Waist Circumference; METS-IR: metabolic score for insulin resistance; AIP: atherogenic index of plasma.

*p < 0.05.

### Association and dose-response relationship between IR surrogate indicators and risk of rapid decline in kidney function and CKD

Table 2 presents the associations between IR surrogate indicators and the risk of rapid decline in kidney function and CKD in middle-aged and older adults without diabetes. The results show that in the analysis of rapid decline in kidney function, both Model 1 and Model 2 indicate a negative correlation between the eGDR and its risk, while LAP, CVAI, TyG index, TyG-WC, and AIP are positively correlated with the risk of rapid decline in kidney function. After adjusting for all confounding factors (Model 3), the association between AIP and the risk of rapid decline in kidney function remained statistically significant. Specifically, for each one SD increase in AIP, the adjusted OR was 1.391 (95% CI: 1.139–1.706). In the analysis of CKD risk, in both Model 1 and Model 2, eGDR was significantly negatively correlated with its risk, while TyG, TyG-BMI, METS-IR, and AIP were significantly positively correlated with CKD risk. After adjusting for all confounders (Model 3), the associations between TyG-BMI, METS-IR, and AIP and CKD risk remained statistically significant. Specifically, for each one standard deviation increase in TyG-BMI, METS-IR, and AIP, the adjusted odds ratios (95% CI) were 1.113 (1.005, 1.204), 1.124 (1.022, 1.219), and 1.560 (1.206, 2.035), respectively.

**Table 2.** Multivariable regression analysis of the eight insulin resistance (IR) surrogate indicators and the risk of rapid decline in kidney function and CKD in non-diabetic middle-aged individuals.

	Rapid decline in kidney function in follow-up						CKD in follow-up					
	Model 1		Model 2		Model 3		Model 1		Model 2		Model 3	
Variable	OR (95%CI)	*p* Value	OR (95%CI)	*p* Value	OR (95%CI)	*p* Value	OR (95%CI)	*p* Value	OR (95%CI)	*p* Value	OR (95%CI)	*p* Value
eGDR	0.854 (0.734,0.997)	0.043^*^	0.854 (0.731,0.999)	0.047^*^	0.957 (0.784,1.157)	0.651	0.677 (0.556,0.828)	<0.001^*^	0.728 (0.595,0.894)	0.002^*^	0.842 (0.683,1.039)	0.108
LAP	1.469 (1.317,1.641)	<0.001^*^	1.491 (1.328,1.682)	<0.001^*^	0.814 (0.662,1.010)	0.051	1.171 (1.001,1.339)	0.034^*^	1.172 (0.982,1.360)	0.068	0.889 (0.692,1.169)	0.361
CVAI	1.349 (1.142,1.595)	<0.001^*^	1.385 (1.160,1.655)	<0.001^*^	1.035 (0.855,1.259)	0.729	1.147 (0.923,1.435)	0.225	1.232 (0.978,1.556)	0.078	0.932 (0.744,1.190)	0.559
TyG	1.771 (1.549,2.022)	<0.001^*^	1.824 (1.583,2.100)	<0.001^*^	1.059 (0.799,1.398)	0.690	1.286 (1.055,1.552)	0.011^*^	1.360 (1.104,1.660)	0.003^*^	0.889 (0.595,1.322)	0.564
TyG-BMI	1.052 (0.927,1.132)	0.246	1.046 (0.906,1.131)	0.344	1.025 (0.775,1.127)	0.721	1.098 (1.002,1.179)	0.012^*^	1.107 (1.011,1.196)	0.009^*^	1.113 (1.005,1.204)	0.010^*^
TyG-WC	1.482 (1.261,1.741)	<0.001^*^	1.546 (1.288,1.943)	<0.001^*^	0.877 (0.725,1.075)	0.877	1.240 (0.998,1.541)	0.053	1.199 (0.953,1.510)	0.123	0.886 (0.694,1.151)	0.348
METS-IR	1.070 (0.970,1.151)	0.079	1.069 (0.960,1.152)	0.102	1.070 (0.869,1.169)	0.245	1.102 (1.009,1.188)	0.009^*^	1.118 (1.023,1.208)	0.004^*^	1.124 (1.022,1.219)	0.005^*^
AIP	1.845 (1.607,2.117)	<0.001^*^	1.923 (1.661,2.228)	<0.001^*^	1.391 (1.139,1.706)	0.001^*^	1.407 (1.158,1.696)	<0.001^*^	1.521 (1.239,1.854)	<0.001^*^	1.560 (1.206,2.035)	0.001^*^

Model 1 was unadjusted.

Model 2 was adjusted for sex, age, education level, marital status, residence, smoking status, alcohol consumption, retired, BMI, and WC.

Model 3 was adjusted for Model 2 + TC, HDL-C, LDL-C, Scr, Cys, SUA, BUN, CRP, hypertension, and heart diseases.

*Abbreviation:* OR: odds ratio; CI: confidence interval; eGDR: estimated glucose disposal rate; LAP, Lipid Accumulation Product; CVAI: Chinese visceral adiposity index; TyG: triglyceride-glucose; TyG-BMI: TyG-body mass index; TyG-WC: TyG-waist circumference; METS-IR: metabolic score for insulin resistance; AIP: atherogenic index of plasma; WC: waist circumference; TC: total cholesterol; HDL-C: high-density lipoprotein cholesterol; LDL-C: low-density lipoprotein cholesterol; Scr: serum creatinine; Cys: Cystatin C; SUA: serum uric acid; BUN: blood urea nitrogen; CRP: C-reactive protein.

*p < 0.05.

**Figure 2.**
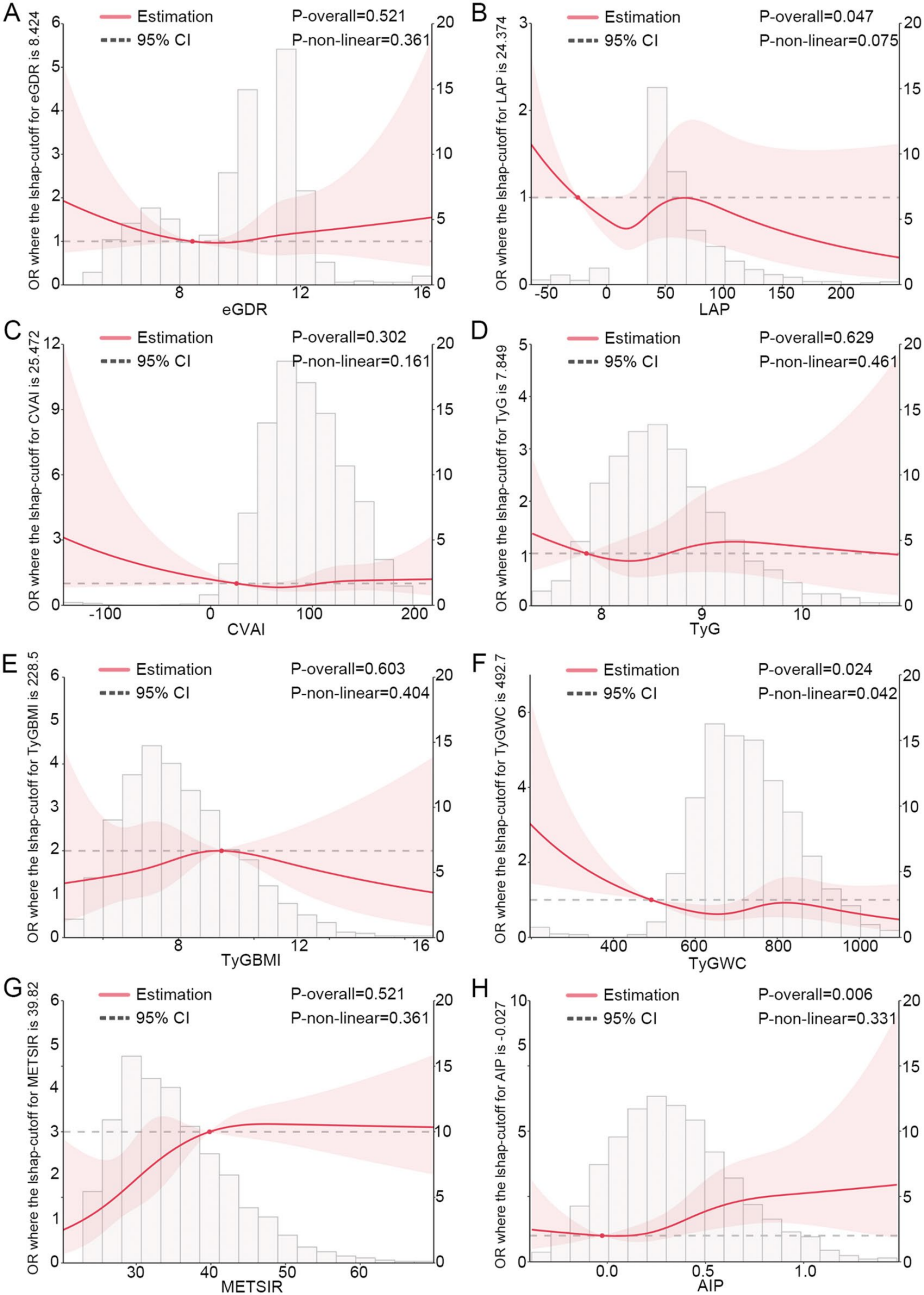
Dose-response relationship between insulin resistance (IR) surrogate indicators and the risk of rapid decline in kidney function. (A) eGDR is the estimated glucose disposal rate; (B) LAP is the lipid accumulation product; (C) CVAI is the Chinese Visceral Adiposity Index; (D) TyG is the triglyceride-glucose index; (E) TyG-BMI is the TyG-body mass index; (F) TyG-WC is the triglyceride-glucose-waist circumference; (G) METS-IR is the metabolic syndrome insulin resistance score; (H) AIP is the Atherogenic Index of Plasma. We fully adjusted the models for sex, age, smoking status, alcohol consumption, marital status, education level, household registration status, retirement status, body mass index (BMI), waist circumference (WC), total cholesterol (TC), low-density lipoprotein cholesterol (LDL-C), creatinine (Scr), cystatin C (Cys), uric acid (SUA), blood urea nitrogen (BUN), C-reactive protein (CRP), hypertension, and heart disease.

**Figure 3.**
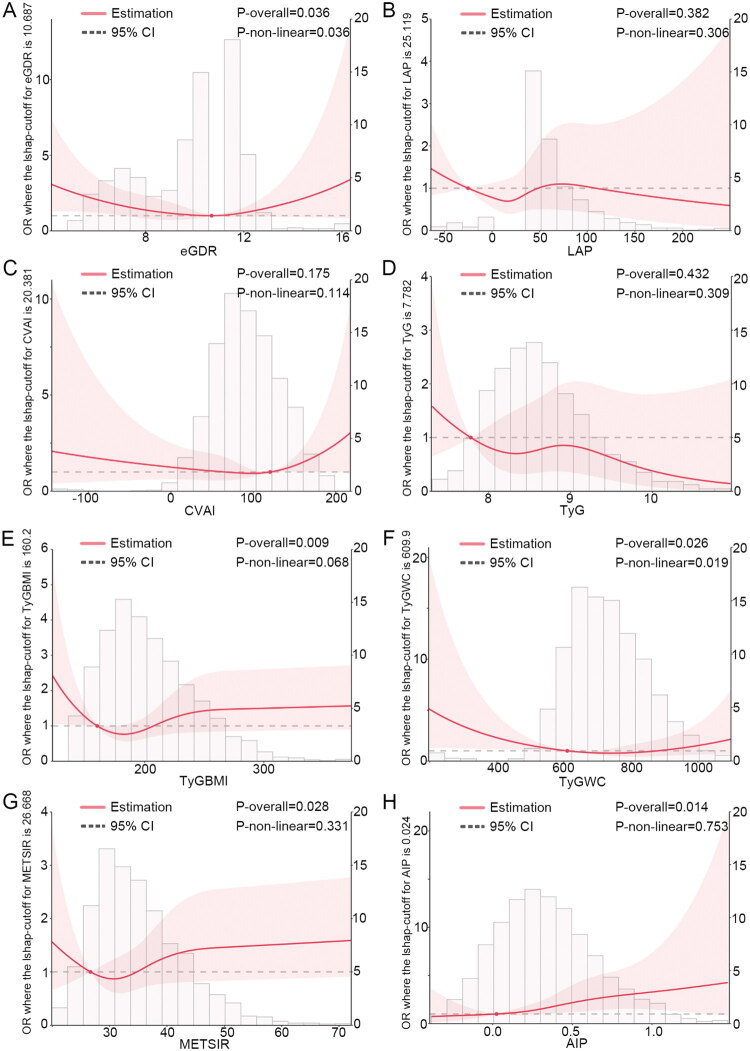
Dose-response relationship between insulin resistance (IR) surrogate indicators and the risk of CKD. (A) eGDR is the estimated glucose disposal rate; (B) LAP is the lipid accumulation product; (C) CVAI is the Chinese visceral adiposity index; (D) TyG is the triglyceride-glucose index; (E) TyG-BMI is the TyG-body mass index; (F) TyG-WC is the triglyceride-glucose-waist circumference; (G) METS-IR is the metabolic syndrome insulin resistance score; (H) AIP is the atherogenic index of plasma. We fully adjusted the models for sex, age, smoking status, alcohol consumption, marital status, education level, household registration status, retirement status, body mass index (BMI), waist circumference (WC), total cholesterol (TC), high-density lipoprotein cholesterol (HDL-C), low-density lipoprotein cholesterol (LDL-C), creatinine (Scr), cystatin C (Cys), uric acid (SUA), blood urea nitrogen (BUN), C-reactive protein (CRP), hypertension, and heart disease.

To explore the dose-response relationship in greater depth, this study used RCS analysis to assess the dose-response relationship between the eight IR surrogate indicators and the risks of rapid decline in kidney function and CKD. For the risk of rapid decline in kidney function (Figure 2), after adjusting for confounding factors, TyG-WC and METS-IR exhibited a nonlinear relationship with the risk (Pnonlinear = 0.042; P nonlinear = 0.032), while eGDR, LAP, CVAI, TyG index, TyG-BMI, and AIP showed a linear relationship with the risk (P nonlinear > 0.05). For CKD risk (Figure 3), after adjusting for confounders, eGDR and TyG-WC showed a nonlinear relationship with the risk (P nonlinear = 0.036; Pnonlinear = 0.019), while LAP, CVAI, TyG index, TyG-BMI, METS-IR, and AIP displayed a linear relationship with CKD risk (P nonlinear > 0.05).

Further, this study categorized the eight IR surrogate indicators into four groups for trend regression analysis (Table S2). For rapid decline in kidney function, after adjusting for relevant confounders, the trend regression analysis results for LAP, TyG-WC, METS-IR, and AIP were significant. The adjusted trend regression OR values were 1.60 (1.35, 1.90), 1.561 (1.28, 1.90), 1.191 (1.001, 1.417), and 1.325 (1.113, 1.578), with the fourth quartile subgroup showing a significant association for each. For CKD, after adjusting for relevant confounders, only the trend regression analysis for AIP was significant, with an adjusted trend regression OR of 1.388 (1.105, 1.745).

### Subgroup analysis to determine the association between IR surrogate indices and the risk of rapid decline in kidney function and CKD

To further explore the relationship between IR surrogate indicators and the risk of rapid decline in kidney function and CKD, this study performed subgroup analyses based on sex and age for participants without diabetes. As shown in Table 3, the results revealed significant differences in the association between IR surrogate indicators and the risk of rapid decline in kidney function and CKD across sex and age groups. For rapid decline in kidney function, the significant association between AIP and its risk was only observed in female and older participants (age ≥ 60 years), with aOR of 1.481 (1.109, 1.978) and 1.409 (1.073, 1.850), respectively. Additionally, CVAI was significantly associated with rapid decline in kidney function in female participants, with an aOR of 1.438 (1.056, 1.957). Interestingly, the study also found a significant negative association between LAP and rapid decline in kidney function in male and older participants, and between TyG-WC and rapid decline in kidney function in male participants. For CKD risk, TyG-BMI and AIP were significantly associated with the risk in male and older participants. Specifically, the aOR for TyG-BMI in males was 1.122 (1.033, 1.219), and 1.101 (1.005, 1.205) in older participants. The aOR for AIP in males was 1.497 (1.017, 2.203), and 1.605 (1.218, 2.115) in older participants. Furthermore, the study also found a significant relationship between METS-IR and CKD risk in older participants, with an aOR of 1.111 (1.007, 1.225).

**Table 3.** Subgroup analysis of the eight insulin resistance (IR) surrogate indicators and the risk of rapid decline in kidney function and CKD in non-diabetic middle-aged individuals.

	Rapid decline in kidney function in follow-up		CKD in follow-up	
Subgroup	aOR (95% CI)	*p*-Value	aOR (95% CI)	*p*-Value
eGDR				
Sex				
Male	1.023 (0.754,1.387)	0.885	0.949 (0.801,1.125)	0.546
Female	0.904 (0.714,1.145)	0.404	1.117 (0.936,1.332)	0.221
Age				
45–60	0.936 (0.712,1.229)	0.633	1.123 (0.940,1.341)	0.203
≥60	0.967 (0.751,1.245)	0.793	0.955 (0.807,1.129)	0.588
LAP				
Sex				
Male	0.500 (0.329,0.762)	0.001^*^	0.871 (0.644,1.179)	0.372
Female	0.941 (0.741,1.194)	0.615	0.894 (0.706,1.132)	0.351
Age				
45–60	0.879 (0.680,1.136)	0.324	0.842 (0.685,1.034)	0.101
≥60	0.649 (0.439,0.961)	0.031^*^	0.941 (0.677,1.308)	0.718
CVAI				
Sex				
Male	0.792 (0.628,1.001)	0.053	0.923 (0.707,1.205)	0.555
Female	1.438 (1.056,1.957)	0.021^*^	0.913 (0.590,1.412)	0.682
Age				
45–60	0.988 (0.742,1.315)	0.933	1.311 (0.599,2.869)	0.498
≥60	1.084 (0.822,1.429)	0.569	0.878 (0.685,1.125)	0.304
TyG				
Sex				
Male	0.925 (0.618,1.384)	0.703	1.168 (0.641,2.129)	0.611
Female	1.105 (0.744,1.642)	0.621	0.657 (0.373,1.158)	0.147
Age				
45–60	1.141 (0.746,1.743)	0.543	0.866 (0.298,2.521)	0.792
≥60	0.928 (0.637,1.350)	0.695	0.969 (0.636,1.478)	0.885
TyG-BMI				
Sex				
Male	0.432 (0.118,1.584)	0.206	1.122 (1.033,1.219)	0.006^*^
Female	1.261 (0.471,3.376)	0.644	0.386 (0.111,1.339)	0.134
Age				
45–60	1.020 (0.775,1.343)	0.886	1.069 (0.665,1.720)	0.782
≥60	0.596 (0.205,1.731)	0.342	1.101 (1.005,1.205)	0.038^*^
TyG-WC				
Sex				
Male	0.654 (0.490,0.874)	0.004^*^	0.909 (0.621,1.330)	0.623
Female	1.061 (0.814,1.384)	0.659	0.925 (0.667,1.283)	0.639
Age				
45–60	0.949 (0.723,1.245)	0.707	1.501 (0.658,3.426)	0.334
≥60	0.791 (0.591,1.059)	0.116	0.857 (0.643,1.143)	0.294
METS-IR				
Sex				
Male	0.983 (0.692,1.396)	0.924	0.864 (0.552,1.354)	0.524
Female	2.411 (1.195,4.862)	0.014^*^	1.149 (0.669,1.972)	0.615
Age				
45–60	1.058 (0.903,1.241)	0.485	1.069 (0.709,1.611)	0.751
≥60	1.060 (0.878,1.280)	0.543	1.111 (1.007,1.225)	0.037^*^
AIP				
Sex				
Male	1.183 (0.895,1.564)	0.237	1.497 (1.017,2.203)	0.041^*^
Female	1.481 (1.109,1.978)	0.008^*^	1.378 (0.960,1.978)	0.082
Age				
45–60	1.306 (0.981,1.739)	0.068	1.088 (0.534,2.215)	0.816
≥60	1.409 (1.073,1.850)	0.014^*^	1.605 (1.218,2.115)	0.001^*^

Subgroup analysis of insulin resistance (IR) surrogate indicators and the risk of rapid kidney function decline and CKD. Each subgroup was adjusted for sex, age, education level, marital status, residence, smoking status, alcohol consumption, retirement status, body mass index (BMI), waist circumference (WC), total cholesterol (TC), low-density lipoprotein cholesterol (LDL-C), serum creatinine (Scr), cystatin C (Cys), serum uric acid (SUA), blood urea nitrogen (BUN), C-reactive protein (CRP), hypertension, and heart diseases: excluding the stratification variables. The odds ratio (OR) corresponding to each 1 standard deviation (SD) increase in the IR surrogate indicators for stroke risk is presented.

*Abbreviations:* CI: confidence interval; eGDR: estimated glucose disposal rate; LAP: lipid accumulation product; CVAI: Chinese Visceral Adiposity Index; TyG: triglyceride-glucose index; TyG-BMI: TyG-body mass index; TyG-WC: triglyceride-glucose-waist circumference; METS-IR: metabolic syndrome insulin resistance score; AIP: Atherogenic Index of Plasma.

*p < 0.05.

### Predictive performance of IR surrogate indicators for rapid decline in kidney function and CKD risk in the overall population and different sex subgroups

**Figure 4.**
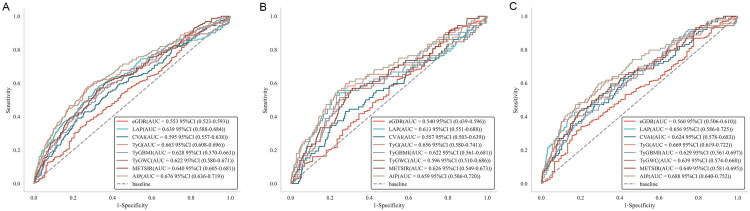
Receiver operating characteristic (ROC) curves of IR surrogate indicators and the risk of rapid decline in kidney functioning different sex groups. (A) Rapid kidney function decline in the general population; (B) Rapid kidney function decline in males; (C) Rapid kidney function decline in females. ROC: receiver operating characteristic; AUC: area under the curve; eGDR: estimated glucose disposal rate; LAP: lipid accumulation product; CVAI: Chinese visceral adiposity index; TyG: triglyceride-glucose index; TyG-BMI: TyG-body mass index; TyG-WC: triglyceride-glucose-waist circumference; METS-IR: metabolic syndrome insulin resistance score; AIP: atherogenic index of plasma.

Table 4 and Figure 4 display the predictive ability of IR surrogate indicators for the risk of rapid decline in kidney function in the overall population as well as in male and female subgroups. In the non-diabetic general population, AIP demonstrated stronger predictive ability for the risk of rapid decline in kidney function compared to other IR surrogate indicators, with the highest area under the ROC curve (AUC = 0.676, 95% CI: 0.638–0.707), followed by TyG index (AUC = 0.663, 95% CI: 0.609–0.707). DeLong test results indicated that the predictive ability of AIP was significantly superior to all other IR surrogate indicators except for TyG index (*p* < 0.05). In both sex subgroups, AIP had the highest AUC, with values of 0.659 (95% CI: 0.586–0.720) in males and 0.688 (95% CI: 0.640–0.752) in females. In males, DeLong tests revealed that AIP was significantly superior to eGDR, CVAI, and TyG-WC in predicting the risk of rapid decline in kidney function (*p* = 0.002; *p* = 0.001; *p* = 0.011). In females, AIP’s predictive ability was significantly superior to eGDR, CVAI, and TyG-BMI (*p* < 0.001; *p* = 0.018; *p* = 0.024). Comparisons between other indicators are provided in Table S3.

**Table 4.** Receiver operating characteristic (ROC) curves of insulin resistance surrogate indicators and the risk of rapid decline in kidney function in non-diabetic middle-aged individuals by sex group.

Group	Variable	AUC (95% CI)	*p* For comparison	Optimal cutoff value	Sensitivity	Specificity	Youden index	Accuracy
All	eGDR	0.553 (0.493–0.607)	<0.001^*^	10.979 (6.960–11.178)	0.368 (0.303–0.893)	0.733 (0.229–0.832)	0.101 (0.060–0.194)	0.046 (0.040–0.052)
	LAP	0.639 (0.591–0.702)	0.029^*^	37.091 (33.254–63.520)	0.584 (0.367–0.674)	0.669 (0.621–0.869)	0.253 (0.212–0.355)	0.955 (0.947–0.961)
	CVAI	0.595 (0.531–0.656)	<0.001^*^	125.087 (92.638–133.392)	0.391 (0.336–0.734)	0.780 (0.527–0.826)	0.172 (0.107–0.285)	0.955 (0.948–0.962)
	TyG	0.663 (0.609–0.707)	0.234	8.900 (8.828–9.183)	0.578 (0.416–0.641)	0.718 (0.680–0.836)	0.296 (0.235–0.366)	0.954 (0.949–0.962)
	TyG-BMI	0.628 (0.587–0.675)	0.006^*^	208.456 (195.848–228.885)	0.590 (0.474–0.705)	0.626 (0.509–0.780)	0.216 (0.167–0.289)	0.954 (0.947–0.961)
	TyG-WC	0.622 (0.563–0.660)	0.005^*^	799.657 (746.401–815.814)	0.491 (0.388–0.645)	0.743 (0.595–0.781)	0.233 (0.160–0.307)	0.954 (0.948–0.959)
	METS-IR	0.640 (0.593–0.687)	0.020^*^	36.319 (35.843–41.638)	0.602 (0.415–0.665)	0.639 (0.610–0.830)	0.241 (0.182–0.323)	0.954 (0.948–0.960)
	AIP	0.676 (0.638–0.707)	Reference	0.500 (0.376–0.649)	0.578 (0.447–0.675)	0.728 (0.600–0.851)	0.306 (0.242–0.370)	0.717 (0.603–0.838)
Male	eGDR	0.540 (0.439–0.596)	0.002^*^	6.832 (6.677–11.765)	0.913 (0.116–0.930)	0.194 (0.156–0.967)	0.107 (0.049–0.230)	0.043 (0.034–0.051)
	LAP	0.613 (0.551–0.688)	0.051	37.151 (31.925–44.765)	0.528 (0.439–0.646)	0.777 (0.726–0.839)	0.305 (0.232–0.421)	0.956 (0.946–0.966)
	CVAI	0.557 (0.503–0.639)	0.001^*^	111.411 (74.409–150.783)	0.431 (0.250–0.730)	0.721 (0.425–0.897)	0.151 (0.115–0.275)	0.956 (0.947–0.964)
	TyG	0.656 (0.580–0.741)	0.823	8.912 (8.635–9.190)	0.556 (0.408–0.724)	0.762 (0.630–0.858)	0.317 (0.225–0.448)	0.957 (0.949–0.963)
	TyG-BMI	0.622 (0.561–0.681)	0.108	214.783 (201.412–227.432)	0.514 (0.399–0.675)	0.750 (0.627–0.836)	0.264 (0.166–0.389)	0.956 (0.948–0.965)
	TyG-WC	0.596 (0.510–0.686)	0.011^*^	775.790 (757.362–800.754)	0.542 (0.436–0.677)	0.723 (0.667–0.782)	0.264 (0.156–0.411)	0.957 (0.949–0.964)
	METS-IR	0.626 (0.549–0.673)	0.088	36.365 (36.365–40.774)	0.569 (0.397–0.667)	0.688 (0.660–0.830)	0.257 (0.149–0.332)	0.956 (0.947–0.965)
	AIP	0.659 (0.586–0.720)	Reference	0.466 (0.360–0.619)	0.597 (0.450–0.738)	0.720 (0.586–0.839)	0.317 (0.221–0.418)	0.731 (0.593–0.824)
Female	eGDR	0.560 (0.506–0.610)	<0.001^*^	10.981 (6.960–11.240)	0.340 (0.259–0.879)	0.798 (0.228–0.872)	0.137 (0.080–0.210)	0.048 (0.038–0.058)
	LAP	0.656 (0.586–0.725)	0.158	64.745 (32.526–73.862)	0.427 (0.336–0.801)	0.830 (0.509–0.876)	0.257 (0.179–0.402)	0.952 (0.944–0.962)
	CVAI	0.624 (0.578–0.683)	0.018^*^	103.833 (98.113–135.267)	0.629 (0.368–0.755)	0.579 (0.521–0.841)	0.208 (0.166–0.323)	0.954 (0.942–0.962)
	TyG	0.669 (0.619–0.722)	0.215	9.156 (8.819–9.431)	0.483 (0.377–0.714)	0.799 (0.633–0.901)	0.282 (0.223–0.385)	0.952 (0.945–0.960)
	TyG-BMI	0.629 (0.561–0.697)	0.024^*^	243.998 (178.520–247.284)	0.360 (0.359–0.953)	0.827 (0.251–0.842)	0.186 (0.126–0.329)	0.951 (0.937–0.962)
	TyG-WC	0.639 (0.574–0.668)	0.078	801.118 (668.947–876.096)	0.506 (0.331–0.933)	0.723 (0.293–0.883)	0.228 (0.163–0.299)	0.953 (0.945–0.960)
	METS-IR	0.649 (0.581–0.695)	0.102	37.260 (36.319–42.991)	0.596 (0.384–0.718)	0.646 (0.595–0.839)	0.241 (0.180–0.340)	0.952 (0.945–0.959)
	AIP	0.688 (0.640–0.752)	Reference	0.500 (0.355–0.763)	0.596 (0.421–0.783)	0.710 (0.532–0.901)	0.306 (0.263–0.429)	0.742 (0.544–0.881)

*Abbreviation*: ROC: receiver operating characteristic; AUC: area under the curve; CI: confidence interval; eGDR: estimated glucose disposal rate; LAP, Lipid Accumulation Product; CVAI: Chinese visceral adiposity index; TyG: triglyceride-glucose; TyG-BMI: TyG-body mass index; TyG-WC: TyG-Waist Circumference; METS-IR: metabolic score for insulin resistance; AIP: atherogenic index of plasma; WC: waist circumference; TC: total cholesterol; LDL-C: low-density lipoprotein cholesterol; Scr: serum creatinine; Cys: Cystatin C; SUA: serum uric acid; BUN: blood urea nitrogen; CRP: C-reactive protein.

*p < 0.05.

**Figure 5.**
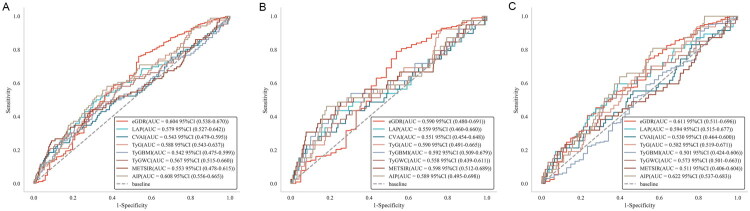
Receiver operating characteristic (ROC) curves of IR surrogate indicators and the risk of CKD in different sex groups. (A) CKD in the general population; (B) CKD in males; (C) CKD in females. ROC: receiver operating characteristic; AUC: area under the curve; eGDR: estimated glucose disposal rate; LAP: lipid accumulation product; CVAI: Chinese visceral adiposity index; TyG: triglyceride-glucose index; TyG-BMI: TyG-body mass index; TyG-WC: triglyceride-glucose-waist circumference; METS-IR: metabolic syndrome insulin resistance score; AIP: atherogenic index of plasma.

Table 5 and Figure 5 show the predictive ability of IR surrogate indicators for CKD risk in the overall population and male and female subgroups. In the non-diabetic general population, AIP demonstrated the strongest predictive ability for CKD risk compared to other IR surrogate indicators, with the highest AUC (AUC = 0.608, 95% CI: 0.549–0.665), followed by eGDR (AUC = 0.604, 95% CI: 0.542–0.669). DeLong test results indicated that AIP’s predictive ability was significantly superior to all other IR surrogate indicators except for TyG index (*p* < 0.05). In the subgroups accrding to sex, AIP had the highest AUC in females, with an AUC of 0.622 (95% CI: 0.537–0.683), while in males, METS-IR had the highest AUC, with an AUC of 0.598 (95% CI: 0.491–0.665). Comparisons between other indicators are presented in Table S4.

**Table 5.** Receiver operating characteristic (ROC) curves of insulin resistance surrogate indicators and the risk of CKD in non-diabetic middle-aged individuals by sex groups.

Group	Variable	AUC (95% CI)	*p* For comparison	Optimal cutoff value	Sensitivity	Specificity	Youden index	Accuracy
All	eGDR	0.604 (0.542–0.669)	<0.001^*^	8.758 (7.096–10.404)	0.760 (0.531–0.873)	0.465 (0.309–0.691)	0.225 (0.163–0.339)	0.024 (0.019–0.029)
	LAP	0.579 (0.525–0.646)	0.029^*^	40.196 (23.038–62.864)	0.488 (0.295–0.719)	0.695 (0.455–0.848)	0.184 (0.120–0.291)	0.976 (0.972–0.982)
	CVAI	0.543 (0.476–0.601)	<0.001^*^	154.584 (72.452–172.189)	0.186 (0.109–0.794)	0.927 (0.326–0.967)	0.113 (0.057–0.215)	0.976 (0.970–0.981)
	TyG	0.588 (0.540–0.641)	0.234	8.760 (8.101–9.052)	0.547 (0.415–0.942)	0.633 (0.193–0.775)	0.179 (0.115–0.265)	0.976 (0.971–0.981)
	TyG-BMI	0.542 (0.482–0.584)	0.006^*^	231.410 (178.866–246.193)	0.349 (0.271–0.793)	0.789 (0.316–0.865)	0.138 (0.056–0.242)	0.975 (0.970–0.980)
	TyG-WC	0.567 (0.507–0.652)	0.005^*^	789.757 (719.125–918.355)	0.442 (0.208–0.690)	0.710 (0.504–0.930)	0.151 (0.104–0.280)	0.976 (0.972–0.980)
	METS-IR	0.553 (0.466–0.607)	0.020^*^	46.361 (38.032–47.323)	0.256 (0.166–0.516)	0.910 (0.697–0.925)	0.165 (0.077–0.253)	0.976 (0.970–0.982)
	AIP	0.608 (0.549–0.665)	Reference	0.412 (0.108–0.577)	0.581 (0.413–0.910)	0.627 (0.249–0.784)	0.209 (0.134–0.311)	0.640 (0.265–0.777)
Male	eGDR	0.590 (0.480–0.691)	0.051	8.531 (6.897–10.377)	0.790 (0.584–0.903)	0.462 (0.312–0.690)	0.252 (0.113–0.415)	0.024 (0.018–0.031)
	LAP	0.559 (0.460–0.660)	0.395	36.127 (23.441–69.406)	0.436 (0.192–0.647)	0.762 (0.579–0.918)	0.198 (0.077–0.358)	0.976 (0.969–0.983)
	CVAI	0.551 (0.454–0.648)	0.396	154.601 (65.068–172.511)	0.231 (0.163–0.829)	0.919 (0.324–0.963)	0.150 (0.077–0.289)	0.976 (0.969–0.983)
	TyG	0.590 (0.491–0.665)	0.950	8.912 (8.098–9.066)	0.462 (0.375–0.968)	0.753 (0.245–0.816)	0.215 (0.137–0.347)	0.976 (0.969–0.983)
	TyG-BMI	0.592 (0.509–0.679)	0.926	214.783 (208.545–245.504)	0.487 (0.299–0.679)	0.744 (0.684–0.894)	0.232 (0.147–0.405)	0.976 (0.967–0.984)
	TyG-WC	0.558 (0.439–0.611)	0.438	914.747 (720.599–943.381)	0.231 (0.113–0.659)	0.933 (0.550–0.956)	0.163 (0.054–0.304)	0.977 (0.971–0.984)
	METS-IR	0.598 (0.512–0.689)	0.767	38.032 (34.983–47.340)	0.487 (0.252–0.718)	0.748 (0.599–0.929)	0.235 (0.164–0.394)	0.976 (0.970–0.982)
	AIP	0.589 (0.495–0.698)	Reference	0.593 (0.468–0.630)	0.462 (0.330–0.667)	0.817 (0.715–0.848)	0.279 (0.148–0.465)	0.801 (0.714–0.839)
Female	eGDR	0.611 (0.511–0.696)	<0.001^*^	8.766 (6.033–10.738)	0.742 (0.404–0.952)	0.468 (0.202–0.862)	0.210 (0.091–0.370)	0.025 (0.018–0.032)
	LAP	0.594 (0.515–0.677)	0.417	40.400 (24.374–97.573)	0.596 (0.248–0.829)	0.613 (0.357–0.927)	0.209 (0.108–0.357)	0.975 (0.969–0.981)
	CVAI	0.530 (0.464–0.600)	0.013^*^	146.219 (71.698–159.577)	0.213 (0.130–0.910)	0.890 (0.226–0.948)	0.103 (0.065–0.243)	0.975 (0.967–0.980)
	TyG	0.582 (0.519–0.671)	0.108	8.674 (8.210–8.965)	0.638 (0.468–0.995)	0.524 (0.205–0.712)	0.162 (0.122–0.333)	0.974 (0.968–0.981)
	TyG-BMI	0.501 (0.424–0.606)	0.001^*^	158.266 (147.416–280.732)	0.912 (0.051–0.960)	0.191 (0.076–0.985)	0.103 (0.029–0.216)	0.026 (0.020–0.032)
	TyG-WC	0.573 (0.501–0.663)	0.236	789.757 (701.511–918.577)	0.489 (0.231–0.748)	0.681 (0.392–0.927)	0.170 (0.094–0.324)	0.975 (0.969–0.982)
	METS-IR	0.511 (0.406–0.604)	0.001^*^	46.361 (28.184–48.516)	0.213 (0.165–0.933)	0.901 (0.157–0.935)	0.113 (0.059–0.281)	0.975 (0.970–0.982)
	AIP	0.622 (0.537–0.683)	Reference	0.279 (0.059–0.461)	0.809 (0.586–1.000)	0.433 (0.171–0.660)	0.241 (0.171–0.376)	0.519 (0.191–0.659)

*Abbreviation:* ROC: receiver operating characteristic; AUC: area under the curve; CI: confidence interval; eGDR: estimated glucose disposal rate; LAP: Lipid Accumulation Product; CVAI: Chinese visceral adiposity index; TyG: triglyceride-glucose; TyG-BMI: TyG-body mass index; TyG-WC: TyG-Waist Circumference; METS-IR: metabolic score for insulin resistance; AIP: atherogenic index of plasma; WC: waist circumference; TC: total cholesterol; LDL-C: low-density lipoprotein cholesterol; Scr: serum creatinine; Cys: Cystatin C; SUA: serum uric acid; BUN: blood urea nitrogen; CRP: C-reactive protein.

*p < 0.05.

### Predictive ability of IR surrogate indicators for rapid decline in kidney function and CKD risk in different sex and age groups

Table 6 shows the predictive ability of IR surrogate indicators for the risk of rapid decline in kidney function across different sex and age groups. The study found that in older males and older females, AIP had the highest area under the ROC curve, with values of 0.640 (95% CI: 0.510–0.731) and 0.747 (95% CI: 0.666–0.839), respectively. DeLong test results showed that in older females, AIP outperformed all other IR surrogate indicators, except for LAP and TyG index, in predicting the risk of rapid decline in kidney function (*p* < 0.05). In older males, AIP’s predictive ability was significantly superior to CVAI (*p* = 0.035). In middle-aged males and middle-aged females, TyG index and METS-IR had the highest AUC values, with 0.707 (95% CI: 0.622–0.794) and 0.640 (95% CI: 0.556–0.707), respectively. AIP was the second highest indicator in both of these groups, with AUC values of 0.702 (95% CI: 0.622–0.834) and 0.639 (95% CI: 0.573–0.726). DeLong test results indicated no significant difference between AIP and the highest AUC indicators in these two groups. Comparisons between other indicators are provided in Table S5. Due to the smaller number of individuals diagnosed with CKD, the sample size for each subgroup was further limited, restricting the establishment of predictive models. Relevant data can be found in Tables S6 and S7. In all sex and age groups, the AUC values of the eight IR surrogate indicators were all above 0.5, indicating that these indicators have certain predictive value for the risk of rapid decline in kidney function and CKD in the non-diabetic population across different age and sex groups.

**Table 6.** Receiver operating characteristic (ROC) curves of insulin resistance (IR) surrogate indicators and the risk of rapid decline in kidney function in non-diabetic middle-aged individuals by sex and age groups.

Group	Variable	AUC (95% CI)	*p* For comparison	Optimal cutoff value	Sensitivity	Specificity	Youden index	Accuracy
Male								
45–60	eGDR	0.566 (0.451–0.667)	0.006^*^	10.209 (6.513–11.439)	0.650 (0.234–0.947)	0.533 (0.140–0.897)	0.183 (0.074–0.358)	0.037 (0.027–0.049)
	LAP	0.642 (0.498–0.760)	0.129	39.052 (24.895–63.869)	0.600 (0.391–0.835)	0.751 (0.573–0.883)	0.351 (0.209–0.565)	0.960 (0.949–0.972)
	CVAI	0.564 (0.462–0.668)	0.003^*^	112.615 (75.454–162.367)	0.500 (0.186–0.787)	0.697 (0.400–0.944)	0.197 (0.090–0.382)	0.961 (0.949–0.974)
	TyG	0.707 (0.622–0.794)	0.776	9.064 (8.635–9.198)	0.633 (0.555–0.824)	0.785 (0.575–0.833)	0.418 (0.305–0.609)	0.963 (0.952–0.974)
	TyG-BMI	0.651 (0.491–0.760)	0.174	215.958 (210.174–230.932)	0.633 (0.384–0.806)	0.710 (0.627–0.812)	0.344 (0.137–0.515)	0.961 (0.951–0.973)
	TyG-WC	0.612 (0.461–0.708)	0.029^*^	795.932 (765.506–919.947)	0.567 (0.223–0.761)	0.729 (0.631–0.928)	0.295 (0.137–0.425)	0.962 (0.948–0.971)
	METS-IR	0.654 (0.559–0.766)	0.133	37.675 (36.559–45.116)	0.633 (0.420–0.874)	0.689 (0.600–0.886)	0.323 (0.203–0.530)	0.962 (0.952–0.972)
	AIP	0.702 (0.622–0.834)	Reference	0.518 (0.343–0.707)	0.667 (0.504–0.867)	0.725 (0.545–0.860)	0.391 (0.305–0.566)	0.746 (0.553–0.851)
≥60	eGDR	0.526 (0.428–0.604)	0.053	6.841 (6.524–11.866)	0.900 (0.110–0.926)	0.214 (0.184–0.981)	0.114 (0.037–0.287)	0.049 (0.036–0.065)
	LAP	0.605 (0.486–0.697)	0.246	34.173 (34.173–46.763)	0.500 (0.315–0.636)	0.795 (0.774–0.880)	0.295 (0.177–0.428)	0.952 (0.941–0.967)
	CVAI	0.559 (0.489–0.639)	0.035^*^	108.569 (64.891–155.726)	0.405 (0.213–0.821)	0.732 (0.370–0.921)	0.136 (0.080–0.336)	0.950 (0.935–0.967)
	TyG	0.632 (0.535–0.723)	0.669	8.860 (8.111–9.615)	0.476 (0.242–0.971)	0.785 (0.269–0.948)	0.262 (0.167–0.427)	0.952 (0.941–0.962)
	TyG-BMI	0.618 (0.528–0.684)	0.491	207.342 (168.913–229.084)	0.500 (0.349–0.918)	0.741 (0.320–0.875)	0.241 (0.163–0.412)	0.952 (0.937–0.964)
	TyG-WC	0.592 (0.520–0.687)	0.136	776.128 (754.998–804.761)	0.500 (0.395–0.665)	0.762 (0.696–0.820)	0.262 (0.186–0.380)	0.952 (0.939–0.964)
	METS-IR	0.620 (0.522–0.698)	0.434	36.365 (29.620–38.430)	0.500 (0.366–0.796)	0.734 (0.384–0.790)	0.234 (0.118–0.430)	0.951 (0.938–0.964)
	AIP	0.640 (0.510–0.731)	Reference	0.449 (0.241–0.598)	0.548 (0.343–0.803)	0.745 (0.483–0.856)	0.293 (0.125–0.443)	0.723 (0.493–0.833)
Female								
45–60	eGDR	0.548 (0.461–0.610)	0.005^*^	11.042 (6.151–11.745)	0.357 (0.151–0.949)	0.792 (0.174–0.983)	0.149 (0.094–0.272)	0.047 (0.036–0.060)
	LAP	0.638 (0.564–0.712)	0.969	46.443 (17.051–81.033)	0.542 (0.359–0.968)	0.687 (0.233–0.899)	0.229 (0.171–0.375)	0.953 (0.943–0.963)
	CVAI	0.622 (0.517–0.679)	0.620	104.271 (98.113–137.249)	0.667 (0.393–0.782)	0.559 (0.493–0.842)	0.226 (0.112–0.348)	0.954 (0.939–0.964)
	TyG	0.633 (0.563–0.704)	0.739	9.369 (8.329–9.500)	0.354 (0.292–0.933)	0.881 (0.311–0.920)	0.235 (0.175–0.370)	0.955 (0.941–0.967)
	TyG-BMI	0.632 (0.563–0.724)	0.824	179.759 (179.759–265.935)	0.979 (0.346–1.000)	0.226 (0.214–0.901)	0.205 (0.179–0.389)	0.954 (0.939–0.968)
	TyG-WC	0.633 (0.554–0.697)	0.869	768.602 (641.850–866.973)	0.604 (0.380–0.981)	0.621 (0.234–0.863)	0.225 (0.178–0.338)	0.954 (0.944–0.966)
	METS-IR	0.640 (0.556–0.707)	0.948	36.446 (28.886–46.749)	0.667 (0.320–1.000)	0.561 (0.177–0.905)	0.228 (0.139–0.376)	0.954 (0.940–0.967)
	AIP	0.639 (0.573–0.726)	Reference	0.754 (0.355–0.763)	0.354 (0.320–0.825)	0.893 (0.503–0.906)	0.247 (0.181–0.415)	0.701 (0.519–0.889)
≥60	eGDR	0.577 (0.521–0.671)	<0.001^*^	10.443 (6.957–11.143)	0.447 (0.280–0.842)	0.732 (0.367–0.909)	0.179 (0.132–0.355)	0.050 (0.036–0.061)
	LAP	0.678 (0.620–0.740)	0.080	67.846 (30.656–104.317)	0.463 (0.331–0.838)	0.859 (0.483–0.954)	0.322 (0.240–0.498)	0.950 (0.937–0.964)
	CVAI	0.625 (0.523–0.686)	0.003^*^	125.156 (66.948–144.890)	0.415 (0.333–0.978)	0.805 (0.206–0.913)	0.219 (0.135–0.370)	0.951 (0.937–0.966)
	TyG	0.713 (0.623–0.801)	0.236	8.819 (8.819–9.401)	0.756 (0.488–0.839)	0.635 (0.621–0.891)	0.391 (0.299–0.552)	0.953 (0.940–0.967)
	TyG-BMI	0.625 (0.545–0.708)	0.003^*^	225.171 (179.715–286.185)	0.463 (0.189–0.920)	0.757 (0.323–0.977)	0.221 (0.145–0.390)	0.951 (0.937–0.964)
	TyG-WC	0.647 (0.553–0.748)	0.032^*^	807.902 (683.280–938.155)	0.537 (0.281–0.892)	0.737 (0.344–0.964)	0.274 (0.198–0.420)	0.955 (0.946–0.969)
	METS-IR	0.658 (0.563–0.745)	0.014^*^	40.038 (33.709–45.589)	0.512 (0.330–0.727)	0.791 (0.534–0.910)	0.303 (0.197–0.482)	0.949 (0.936–0.961)
	AIP	0.747 (0.666–0.839)	Reference	0.504 (0.433–0.722)	0.707 (0.505–0.862)	0.728 (0.626–0.887)	0.436 (0.312–0.601)	0.756 (0.638–0.873)

*Abbreviation:* ROC: receiver operating characteristic; AUC: area under the curve; CI: confidence interval; eGDR: estimated glucose disposal rate; LAP: Lipid Accumulation Product; CVAI: Chinese visceral adiposity index; TyG: triglyceride-glucose; TyG-BMI: TyG-body mass index; TyG-WC: TyG-Waist Circumference; METS-IR: metabolic score for insulin resistance; AIP: atherogenic index of plasma; WC: waist circumference; TC: total cholesterol; LDL-C: low-density lipoprotein cholesterol; Scr: serum creatinine; Cys: Cystatin C; SUA: serum uric acid; BUN: blood urea.

*p < 0.05.

## Discussion

Rapid decline in kidney function and CKD is recognized as major public health issues with severe consequences, placing significant burdens on both individuals and society. IR is considered one of the key risk factors for rapid decline in kidney function and CKD. Therefore, developing a rapid and easy-to-use detection method for the early identification and intervention of rapid decline in kidney function and CKD is crucial. This study evaluated the predictive ability of eight surrogate markers of IR for the risk of rapid decline in kidney function and CKD in the Chinese middle-aged and elderly non-diabetic population. Our results showed that, after rigorously adjusting for relevant confounding factors, TyG-BMI and METS-IR were significantly associated with CKD risk, while AIP was significantly associated not only with CKD risk but also with rapid decline in kidney function. All markers demonstrated predictive value for the risk of rapid decline in kidney function and CKD in the Chinese middle-aged and elderly population with impaired glucose metabolism. Among these, AIP showed significant potential in predicting kidney disease risk.

IR is a pathological condition driven by both genetic and environmental factors, commonly observed in individuals with diabetes as well as in those with non-diabetic kidney disease [43]. IR promotes renal function decline and the progression of CKD through mechanisms such as glomerular hypertension, inflammation, oxidative stress, and lipotoxicity, independently of hyperglycemia [44–47]. Consistent evidence indicates that IR is independently associated with renal function decline in non-diabetic populations [43,48–50]. Most studies currently assess IR using the homeostatic model assessment of insulin resistance (HOMA-IR); however, this measure relies on fasting insulin testing, which limits its clinical utility due to procedural complexity and high cost. To overcome these limitations, researchers have recently focused on developing surrogate markers of IR based on its typical clinical features—such as hyperglycemia, dyslipidemia, hypertension, and obesity—which can be combined into composite indices that provide a more convenient assessment of individual IR [51]. For example, the AIP, introduced by Dobiasova and Frohlich in 2001 [52], not only reflects the ratio of pro-atherogenic lipids to protective lipids in plasma but also indicates the size and esterification rate of high-density HDL-C particles. AIP is closely associated with small dense low-density lipoprotein cholesterol (sdLDL-C) levels, which readily convert into oxidized LDL (oxLDL) [53]. OxLDL serves as a key mediator of endothelial dysfunction and oxidative stress and can be taken up by various renal cell types, contributing to glomerulosclerosis and interstitial fibrosis, thereby accelerating the progression of CKD [54–56]. Meanwhile, lipotoxicity leads to excessive lipid accumulation in glomerular endothelial cells, mesangial cells, and podocytes, triggering mitochondrial dysfunction, oxidative stress, and inflammatory responses, which ultimately damage the filtration barrier and promote proteinuria formation [57–59]. Lipid deposition in renal tubular epithelial cells activates inflammatory and fibrotic pathways, promoting tubulointerstitial injury, which ultimately contributes to the onset and progression of renal function impairment [60,61]. Previous studies have shown that elevated AIP levels are associated with an increased risk of CKD in adults with metabolic disorders [62]. In particular, the risk of diabetic kidney disease (DKD) significantly increases with higher AIP values, suggesting that AIP could be a biomarker for early renal dysfunction [63]. Moreover, Smajic et al. reported that AIP is an independent positive predictor of the decline in eGFR in kidney disease patients, and they also found an association between AIP and CKD [64]. Our findings are consistent with these results, as AIP was significantly associated with both CKD and rapid decline in kidney function after adjustment, showing strong correlation and predictive power across all age and sex groups.

As for the TyG index, which is based on TG and FPG, it has been widely used to predict diabetes and cardiovascular diseases. Numerous studies have confirmed an association between the TyG index and CKD as well as renal disease [13,65]. Although in our study, after strict adjustment for confounding factors, the TyG index did not show a significant relationship with rapid decline in kidney function and CKD, its predictive value was similar to that of AIP in the overall non-diabetic population as well as in different age and sex subgroups. This suggests that the TyG index remains an important screening factor. Additionally, obesity, particularly visceral obesity, is the leading cause of IR [66,67]. Common indicators for measuring visceral obesity include BMI, waist circumference, hip circumference, and waist-to-hip ratio. Some studies suggest that TyG-BMI and TyG-WC perform better than TyG index in predicting IR [68]. By integrating the TyG index with BMI and WC, a more comprehensive perspective on IR assessment can be provided. In our study, TyG-BMI showed a significant positive relationship with CKD in the non-diabetic population. Previous studies have also found that TyG-BMI is associated with kidney dysfunction in T2DM patients [69] and may be an independent risk factor for CKD in the general population. Our findings further extend this relationship to the non-diabetic middle-aged and elderly population and its predictive value for kidney disease. Moreover, Liu’s study found that TyG-WC had the highest predictive value for rapid decline in kidney function across the entire middle-aged and elderly population [14], but this result is not consistent with our findings in the non-diabetic middle-aged and elderly population. Although TyG-WC demonstrated good predictive value in our study, it was not the best predictor.

The METS-IR is a novel, non–insulin-dependent metabolic assessment tool developed based on routine biochemical and anthropometric parameters [70,71]. The results of this study demonstrated a significant positive association between METS-IR and the risk of CKD. Current research on the association between METS-IR and CKD remains limited; consistent with our findings, Lin et al. reported that higher METS-IR levels were associated with an increased incidence of CKD in a Chinese population [72]. A cohort study involving nearly 30,000 non-diabetic participants further found that individuals in the highest quartile of METS-IR had a significantly higher risk of developing CKD (OR: 2.360, 95% CI: 1.594–3.493), and that METS-IR had a moderate predictive ability for CKD (AUC = 0.681, 95% CI: 0.671–0.691) [47]. Moreover, findings from the NHANES study suggested that CKD risk increases significantly when METS-IR exceeds a certain threshold [73]. However, Seyyed et al. in a study conducted among Middle Eastern and North African populations, did not observe a significant association between METS-IR and CKD [74], suggesting that its predictive value may vary across populations and requires further validation. Exploratory subgroup analyses indicated that the positive association between METS-IR and CKD was more pronounced in individuals aged over 60 years. This may be attributed to age-related renal function decline and unfavorable changes in body composition—such as sarcopenia and increased visceral adiposity—which amplify the metabolic damage induced by IR represented by METS-IR, thereby strengthening its association with CKD [44,75]. Furthermore, in the non-diabetic population, this study did not detect a significant sex-specific association between METS-IR and CKD, whereas Lin et al. reported a stronger association among women. Such discrepancies may result from population heterogeneity and limited statistical power due to the sample size of the present study [72]. Although the present study suggests that METS-IR has certain predictive value for CKD, current evidence remains limited. Future large-scale prospective studies are warranted to further validate its clinical applicability and elucidate the underlying mechanistic pathways.

CVAI is a reliable indicator for assessing visceral fat distribution in the Chinese population, and previous studies have focused on its use in evaluating the risk of kidney disease. Xu et al.’s study demonstrated a negative correlation between CVAI and eGFR in the Chinese population, with the AUC for CVAI being higher than that of other obesity indicators. This was particularly pronounced in females (AUC = 0.74; 95% CI: 0.71–0.76), while in males, the AUC was 0.58 (95% CI: 0.50–0.60) [76]. Similarly, Kim et al. found that CVAI was significantly associated with a high prevalence of CKD in the Korean population, with the AUC in females (AUC = 0.827) significantly higher than in males (AUC = 0.657) [77]. Additionally, Liu’s study found that CVAI had the highest predictive value for the progression to CKD in the entire middle-aged and elderly population [14]. However, the results of this study are not entirely consistent with these previous studies. In the non-diabetic population, CVAI did not demonstrate significant predictive value for rapid decline in kidney function and CKD, either across different sex or age groups. Regarding LAP, Yan et al. reported a positive correlation between LAP quartiles and CKD risk in women under specific conditions [78]. However, Su et al. found that high LAP levels have a protective effect on kidney function, a finding that partially aligns with our results [79]. In this study, after adjustment, LAP was associated with rapid decline in kidney function and CKD in non-diabetic males and older populations. This difference may be due to the distinct physiological and pathological mechanisms of IR across different populations. In non-diabetic males and the elderly, higher LAP levels may reflect relatively better fat storage capacity, thereby reducing ectopic fat deposition in non-adipose tissues such as the kidneys, which in turn may protect kidney function.

In the overall population, this study found that AIP outperformed other IR surrogate markers in predicting the risk of rapid decline in kidney function and CKD. The calculation of the AIP relies solely on two basic parameters from routine lipid testing—TG and HDL-C—which makes it highly suitable for primary healthcare settings and large-scale population screening, providing an efficient and cost-effective tool for the early identification of individuals at high risk of kidney injury [80]. In addition, dynamic changes in AIP can effectively reflect the efficacy of lifestyle interventions, making it a valuable monitoring indicator in health management [81,82]. Building upon this finding, exploratory subgroup analyses were conducted to preliminarily investigate whether these associations differed by sex and age group. The analyses revealed that, when stratified by sex and age, AIP demonstrated a stronger predictive ability for rapid decline in kidney function in older males and females compared to all other IR markers, except for LAP and TyG index. In contrast, in middle-aged females, the predictive ability of AIP was generally similar to that of other IR surrogate markers for the risk of rapid decline in kidney function. Moreover, in middle-aged and elderly males, AIP and TyG index showed stronger predictive ability for the risk of rapid decline in kidney function. This discrepancy may be attributed to differences in fat distribution, hormone levels, and metabolic changes between sex, as well as metabolic functional differences between middle-aged and elderly individuals. Therefore, when conducting kidney disease risk assessments, it is essential not only to consider the overall effectiveness of these markers but also to pay attention to their performance variations across different sex and age groups to enable personalized health management and risk prediction. Additionally, due to the lower number of CKD cases, the reliability of the predictive and subgroup analysis results should be interpreted with caution, and further validation with larger cohorts is needed.

Based on nationally representative prospective cohort data from the CHARLS, this study systematically evaluated the predictive value of eight surrogate markers of IR for rapid renal function decline and CKD risk among middle-aged and older adults without diabetes in China. Compared with previous CHARLS-based studies, this analysis focused exclusively on non-diabetic individuals, thereby eliminating the direct confounding effects of diabetes on renal function [14,27,28]. This approach clearly revealed the independent association between IR per se and renal outcomes, and identified AIP as the most effective predictive indicator in this population. Methodologically, in addition to the six indicators examined in previous studies, we incorporated eGDR and METS-IR, thereby establishing a more comprehensive IR assessment framework and providing new insights into the early identification and prevention of kidney disease risk. By controlling for potential confounders and performing subgroup analyses, this study ensured the robustness of the results while revealing population heterogeneity in the predictive performance of different IR markers.

## Limitations

However, this study has some limitations. First, it is an observational analysis. Although we have adjusted for a range of confounding factors, residual confounding cannot be entirely eliminated. Furthermore, the CHARLS dataset does not record acute kidney injury (AKI) events, so participants with possible AKI events were neither excluded nor specifically adjusted for in our analysis. Additionally, the sample size of those who eventually developed CKD was relatively small, which may lead to insufficient statistical power, thus limiting the accurate assessment of the relationship between insulin resistance surrogate markers and CKD risk. This limitation was particularly evident in the exploratory subgroup analyses stratified by sex and age, where the results should be interpreted with considerable caution and warrant further confirmation in studies with larger sample sizes. Second, kidney function was only assessed at baseline and exit visits. The lack of more frequent assessments may affect the accurate capture of changes in kidney function over time. Moreover, during the study period, we were unable to control for the treatments patients received, which could further influence the interpretation of the results. Another major limitation is the lack of time-to-event analysis. Without considering the time factor, we could not fully assess its impact on the relationship between insulin resistance surrogate markers and the risk of rapid decline in kidney function and CKD. Therefore, future studies should consider incorporating time-to-event analysis to more comprehensively evaluate the impact of insulin resistance surrogate markers on kidney disease risk. Additionally, the medical history of conditions such as diabetes and hypertension partly relied on self-reported data, which may affect the accuracy and reliability of the data. While we adjusted for and controlled known major confounders, the potential influence of unknown factors, such as genetics, diet, physical activity, and environmental changes, cannot be ruled out. Finally, this study focused on the middle-aged and elderly non-diabetic population in China, and whether the findings can be generalized to populations in other countries requires further investigation and validation.

## Supplementary Material

Supplementary materials.docx

## Data Availability

The data underlying this article will be provided by the corresponding author on reasonable request.
