# Supplementary material for: Comparing the associations of eight surrogate markers of insulin resistance with rapid decline in kidney function and the incidence of CKD in Chinese middle-aged and elderly non-diabetic population
Source: Ren Fail. 2026 Apr 7;48(1):2633853. doi: 10.1080/0886022X.2026.2633853 (PMC13059037; doi:10.1080/0886022X.2026.2633853)
Supplement: Supplementary materials.docx [file IRNF_A_2633853_SM9376.docx]

**Comparing the Associations of Eight Surrogate Markers of Insulin Resistance with Rapid Decline in Kidney Function and the Incidence of CKD in Chinese Middle-aged and Elderly Non-diabetic Population: A Nationwide Longitudinal Cohort Study**

Wei-Zhen Tang ^a,b,¶^, Zhi-Yong Xiang ^a,¶^, Qin-Yu Cai ^a,b^, Hong-Yu Xu ^b^, Zhi-Jian Zhou ^b^, Ting-He Sheng ^a^, Xu-Zhang ^b^, Jun Ding ^a^, Tai-Hang Liu ^b^, Fei Han ^a,c^, Peng Guo^a,*^

**Affiliations:**

^a^ The Third Affiliated Hospital of Chongqing Medical University, Chongqing, 401120, PR China

^b^ Department of Bioinformatics, School of Basic Medical Sciences, Chongqing Medical University, Chongqing, 400016, PR China.

^c^ Department of Health Toxicology, School of Public Health, Chongqing Medical University, Chongqing 400016, PR China

***Corresponding authors:**

Peng Guo ([doctorguopeng@163.com](mailto:doctorguopeng@163.com)). The Third Affiliated Hospital of Chongqing Medical University, No.1, Shuanghu Branch Road, Huixing Street, Yubei District, Chongqing, 401120, China. Tel.: +86 023 60353088.

¶ **W.Z. Tang and Z.Y. Xiang contributed equally to this work.**

**Table S1.** Conduct Variance Inflation Factor (VIF) detection for all variables.

| **Variables** | **Variance Inflation Factor** |
| --- | --- |
| Sex | 2.500271 |
| Age | 1.249417 |
| Education level | 1.321636 |
| Marital status | 1.066987 |
| Residence | 1.520514 |
| Smoking Status | 1.606913 |
| Alcohol consumption | 1.338706 |
| Retired | 1.476301 |
| BMI | 1.019782 |
| WC | 1.148391 |
| TC | 4.088797 |
| HDL-C | 1.233117 |
| LDL-C | 3.672073 |
| Scr | 1.933158 |
| Cys | 1.438787 |
| SUA | 1.542064 |
| BUN | 1.098119 |
| CRP | 1.019434 |
| Hypertension | 1.752314 |
| Heart diseases | 2.108965 |

**Abbreviation:** BMI, Body Mass Index; WC, Waist Circumference; TC, Total Cholesterol; HDL-C, High-Density Lipoprotein Cholesterol; LDL-C, Low-Density Lipoprotein Cholesterol; Scr, Serum Creatinine; Cys, Cystatin C; SUA, Serum Uric Acid; BUN, Blood Urea Nitrogen; CRP, C-Reactive Protein.

**Table S2.** The multivariable trend regression analysis of eight insulin resistance (IR) surrogate markers and their association with rapid decline in kidney function and CKD risk.

| **EC** | **Rapid decline of kidney function** | | | | **CKD** | | | |
| --- | --- | --- | --- | --- | --- | --- | --- | --- |
|  | **Unadjusted Model** | | **Adjusted Model** | | **Unadjusted Model** | | **Adjusted Model** | |
|  | **OR (95% CI)** | **p-value** | **aOR (95% CI)** | **p-value** | **OR (95% CI)** | **p-value** | **aOR (95% CI)** | **p-value** |
| **eGDR** |  |  |  |  |  |  |  |  |
| Q 1 | Reference |  | Reference |  | Reference |  | Reference |  |
| Q 2 | 0.911(0.599,1.387) | 0.664 | 0.780(0.483,1.259) | 0.309 | 0.364(0.199,0.663) | 0.001* | 0.503(0.268,0.943) | 0.032* |
| Q 3 | 0.803(0.521,1.238) | 0.321 | 0.907(0.551,1.492) | 0.700 | 0.339(0.183,0.628) | 0.001* | 0.591(0.307,1.140) | 0.117 |
| Q 4 | 0.612(0.384,0.975) | 0.039* | 0.841(0.488,1.452) | 0.535 | 0.414(0.233,0.735) | 0.003* | 0.74490.395,1.403) | 0.361 |
| P for trend | 0.857(0.743,0.988) | 0.034* | 0.959(0.804,1.143) | 0.638 | 0.709(0.580,0.866) | 0.001* | 0.880(0.711,1.088) | 0.237 |
| **LAP** |  |  |  |  |  |  |  |  |
| Q 1 | Reference |  | Reference |  | Reference |  | Reference |  |
| Q 2 | 0.852(0.493,1.484) | 0.565 | 0.891(0.501,1.584) | 0.696 | 0.990(0.739,1.365) | 0.981 | 1.070(0.775,1.498) | 0.685 |
| Q 3 | 1.223(0.736,2.037) | 0.444 | 1.366[0.793,2.345) | 0.274 | 0.903(0.658,1.246) | 0.504 | 0.951(0.664,1.342) | 0.751 |
| Q 4 | 2.821(1.813,4.414) | <0.001* | 3.384(1.99f,5.730) | <0.001* | 1.054(0.770,1.435) | 0.770 | 1.130(0.784,1.655) | 0.506 |
| P for trend | 1.514(1.304,1.750) | <0.001* | 1.603(1.358,1.907) | <0.001* | 1.001(0.913,1.112) | 0.935 | 1.033(0.918,1.161) | 0.672 |
| **CVAI** |  |  |  |  |  |  |  |  |
| Q 1 | Reference |  | Reference |  | Reference |  | Reference |  |
| Q 2 | 1.034(0.616,1.739) | 0.898 | 0.949(0.550,1.640) | 0.853 | 1.284(0.688,2.396) | 0.433 | 1.495(0.777,2.875) | 0.228 |
| Q 3 | 1.324(0.809,2.167) | 0.264 | 1.029(0.598,1.773) | 0.917 | 0.887(0.449,1.750) | 0.729 | 1.064(0.518,2.184) | 0.866 |
| Q 4 | 2.299(1.467,3.602) | <0.001* | 1.322(0.784,2.230) | 0.295 | 1.630(0.898,2.957) | 0.108 | 1.690(0.886,3.226) | 0.111 |
| P for trend | 1.351(1.167,1.564) | <0.001* | 1.108(0.935,1.314) | 0.236 | 1.132[0.934,1.373] | 0.206 | 1.140(0.929,1.399) | 0.208 |
| **TyG** |  |  |  |  |  |  |  |  |
| Q 1 | Reference |  | Reference |  | Reference |  | Reference |  |
| Q 2 | 1.621(0.920,2.856) | 0.095 | 1.540(0.841,2.817) | 0.162 | 1.203(0.602,2.402) | 0.601 | 0.954(0.462,1.970) | 0.898 |
| Q 3 | 1.309(0.725,2.363) | 0.371 | 1.078(0.563,2.063) | 0.821 | 1.410(0.722,2.753) | 0.315 | 0.949(0.453,1.988) | 0.890 |
| Q 4 | 4.472(2.719,7.355) | <0.001* | 1.689(0.855,3.339) | 0.132 | 2.173(1.169,4.043) | 0.014* | 1.025(0.436,2.407) | 0.955 |
| P for trend | 1.662(1.422,1.942) | <0.001* | 1.128(0.909,1.400) | 0.274 | 1.300(1.067,1.582) | 0.009* | 1.006(0.765,1.324) | 0.965 |
| **TyG-BMI** |  |  |  |  |  |  |  |  |
| Q 1 | Reference |  | Reference |  | Reference |  | Reference |  |
| Q 2 | 1.518(0.878,2.624) | 0.136 | 1.246(0.696,2.232) | 0.459 | 0.853(0.451,1.613) | 0.625 | 0.871(0.444,1.706) | 0.686 |
| Q 3 | 1.760(1.032,3.001) | 0.038* | 1.095(0.594,2.017) | 0.771 | 0.806(0.422,1.538) | 0.513 | 0.684(0.334,1.400) | 0.299 |
| Q 4 | 3.262(1.998,5.325) | <0.001* | 1.292(0.677,2.464) | 0.437 | 1.442(0.819,2.539) | 0.205 | 1.087(0.518,2.283) | 0.825 |
| P for trend | 1.472(1.267,1.710) | <0.001* | 1.064(0.868,1.305) | 0.550 | 1.132(0.934,1.373) | 0.206 | 1.005(0.785,1.288) | 0.968 |
| **TyG-WC** |  |  |  |  |  |  |  |  |
| Q 1 | Reference |  | Reference |  | Reference |  | Reference |  |
| Q 2 | 1.159(0.68,1.98) | 0.587 | 1.200(0.69,2.08) | 0.513 | 0.75(0.538,1.037) | 0.082 | 0.77(0.55,1.09) | 0.137 |
| Q 3 | 1.239(0.73,2.10) | 0.424 | 1.407(0.80,2.49) | 0.240 | 1.00(0.74,1.36) | 1.000 | 1.05(0.74,1.48) | 0.804 |
| Q 4 | 2.967(1.88,4.69) | <0.001* | 3.734(2.06,6.78) | <0.001* | 1.05(0.77,1.42) | 0.762 | 1.10(0.73,1.67) | 0.645 |
| P for trend | 1.472(1.27,1.71) | <0.001* | 1.561(1.28,1.90) | <0.001* | 1.04(0.94,1.15) | 0.406 | 1.05(0.92,1.21) | 0.441 |
| **METS-IR** |  |  |  |  |  |  |  |  |
| Q 1 | Reference |  | Reference |  | Reference |  | Reference |  |
| Q 2 | 1.247(0.730,2.130) | 0.419 | 1.257(0.716,2.206) | 0.426 | 0.552(0.285,1.070) | 0.078 | 0.642(0.321,1.282) | 0.209 |
| Q 3 | 1.501(0.896,2.515) | 0.123 | 1.342(0.769,2.344) | 0.301 | 0.674(0.361,1.257) | 0.214 | 0.843(0.416,1.705) | 0.634 |
| Q 4 | 2.860(1.791,4.569) | <0.001* | 1.739(1.005,3.009) | 0.048* | 1.206(0.703,2.067) | 0.497 | 1.541(0.746,3.184) | 0.242 |
| P for trend | 1.438(1.239,1.669) | <0.001* | 1.191(1.001,1.417) | 0.048* | 1.090(0.899,1.320) | 0.381 | 1.158(0.903,1.486) | 0.247 |
| AIP |  |  |  |  |  |  |  |  |
| Q 1 | Reference |  | Reference |  | Reference |  | Reference |  |
| Q 2 | 0.910(0.500,1.656) | 0.757 | 1.062(0.569,1.985) | 0.850 | 0.999(0.496,2.010) | 0.997 | 0.820(0.394,1.707) | 0.596 |
| Q 3 | 1.635(0.964,2.776) | 0.068 | 1.637(0.931,2.878) | 0.087 | 1.128(0.571,2.226) | 0.729 | 1.038(0.504,2.141) | 0.918 |
| Q 4 | 3.721(2.317,5.975) | <0.001* | 2.174(1.261,3.748) | 0.005* | 2.300(1.267,4.177) | 0.006* | 2.447(1.252,4.783) | 0.009* |
| P for trend | 1.683(1.439,1.968) | <0.001* | 1.325(1.113,1.578) | 0.002* | 1.353(1.110,1.651) | 0.003* | 1.388(1.105,1.745) | 0.005* |

**Abbreviation:** eGDR, estimated glucose disposal rate; LAP，Lipid Accumulation Product；CVAI, Chinese visceral adiposity index; TyG, triglyceride-glucose; TyG-BMI, TyG-body mass index; TyG-WC, TyG-Waist Circumference; METS-IR, metabolic score for insulin resistance; AIP, atherogenic index of plasma;

*p < 0.05

**Table S3.** DeLong test p-value table for the insulin resistance surrogate markers and rapid decline in kidney function across different gender groups.

| **name** | **eGDR** | **LAP** | **CVAI** | **TyG** | **TyG-BMI** | **TyG-WC** | **METSIR** | **AIP** |
| --- | --- | --- | --- | --- | --- | --- | --- | --- |
| **All** |  |  |  |  |  |  |  |  |
| eGDR |  | <0.001* | 0.001 | <0.001* | <0.001* | <0.001* | <0.001* | <0.001* |
| LAP | <0.001* |  | 0.001* | 0.166 | 0.528 | 0.024* | 0.979 | 0.029* |
| CVAI | 0.001* | 0.001* |  | 0.002* | 0.049* | 0.021* | 0.006* | <0.001* |
| TyG | <0.001* | 0.166 | 0.002* |  | 0.034* | 0.027* | 0.180 | 0.234 |
| TyGBMI | <0.001* | 0.528 | 0.049* | 0.034* |  | 0.761 | 0.074 | 0.006* |
| TyG-WC | <0.001* | 0.024* | 0.021* | 0.027* | 0.761 |  | 0.333 | 0.005* |
| METSIR | <0.001* | 0.979 | 0.006* | 0.180 | 0.074 | 0.333 |  | 0.020* |
| AIP | <0.001* | 0.029* | <0.001* | 0.234 | 0.006* | 0.005 | 0.020* |  |
| **Male** |  |  |  |  |  |  |  |  |
| eGDR |  | 0.036* | 0.166 | 0.002* | 0.012* | 0.060* | 0.009* | 0.002* |
| LAP | 0.036* |  | <0.001* | 0.061 | 0.726 | 0.010* | 0.617 | 0.051 |
| CVAI | 0.166 | <0.001* |  | 0.002* | 0.016* | 0.004* | 0.007* | 0.001* |
| TyG | 0.002* | 0.061 | 0.002* |  | 0.117 | 0.011* | 0.181 | 0.823 |
| TyG-BMI | 0.012* | 0.726 | 0.016* | 0.117 |  | 0.289 | 0.685 | 0.108 |
| TyG-WC | 0.060 | 0.010* | 0.004* | 0.011* | 0.289 |  | 0.233 | 0.011* |
| METSIR | 0.009* | 0.617 | 0.007* | 0.181 | 0.685 | 0.233 |  | 0.088 |
| AIP | 0.002* | 0.051 | 0.001* | 0.823 | 0.108 | 0.011* | 0.088 |  |
| **Female** |  |  |  |  |  |  |  |  |
| eGDR |  | <0.001* | 0.001* | <0.001* | <0.001* | 0.001* | <0.001* | <0.001* |
| LAP | <0.001* |  | 0.107 | 0.611 | 0.292 | 0.098 | 0.788 | 0.158 |
| CVAI | 0.001* | 0.107 |  | 0.145 | 0.797 | 0.405 | 0.226 | 0.018* |
| TyG | <0.001* | 0.611 | 0.145 |  | 0.123 | 0.291 | 0.464 | 0.215 |
| TyG-BMI | <0.001* | 0.292 | 0.797 | 0.123 |  | 0.676 | 0.032* | 0.024* |
| TyG-WC | 0.001* | 0.098 | 0.405 | 0.291 | 0.676 |  | 0.681 | 0.078 |
| METSIR | <0.001* | 0.788 | 0.226 | 0.464 | 0.032* | 0.681 |  | 0.102 |
| AIP | <0.001* | 0.158 | 0.018* | 0.215 | 0.024* | 0.078 | 0.102 |  |

**Abbreviation:** eGDR, estimated glucose disposal rate; LAP，Lipid Accumulation Product；CVAI, Chinese visceral adiposity index; TyG, triglyceride-glucose; TyG-BMI, TyG-body mass index; TyG-WC, TyG-Waist Circumference; METS-IR, metabolic score for insulin resistance; AIP, atherogenic index of plasma;

*p < 0.05

**Table S4.** DeLong test p-value table for the insulin resistance surrogate markers and CKD across different gender groups.

| **name** | **eGDR** | **LAP** | **CVAI** | **TyG** | **TyG-BMI** | **TyG-WC** | **METSIR** | **AIP** |
| --- | --- | --- | --- | --- | --- | --- | --- | --- |
| **All** |  |  |  |  |  |  |  |  |
| eGDR |  | 0.005* | 0.023* | <0.001* | 0.017* | 0.009* | 0.011* | <0.001* |
| LAP | 0.005* |  | 0.025* | 0.723 | 0.138 | 0.290 | 0.318 | 0.244 |
| CVAI | 0.023* | 0.025* |  | 0.100 | 0.982 | 0.037 | 0.652 | 0.020* |
| TyG | <0.001* | 0.723 | 0.100 |  | 0.034* | 0.427 | 0.124 | 0.204 |
| TyG-BMI | 0.017* | 0.138 | 0.982 | 0.034 |  | 0.248 | 0.275 | 0.013* |
| TyG-WC | 0.009* | 0.290 | 0.037* | 0.427 | 0.248 |  | 0.541 | 0.154 |
| METSIR | 0.011* | 0.318 | 0.652 | 0.124 | 0.275 | 0.541 |  | 0.016* |
| AIP | <0.001* | 0.244 | 0.020* | 0.204 | 0.013* | 0.154 | 0.016* |  |
| **Male** |  |  |  |  |  |  |  |  |
| eGDR |  | 0.152 | 0.171 | 0.044* | 0.064 | 0.155 | 0.056 | 0.051 |
| LAP | 0.152 |  | 0.677 | 0.361 | 0.288 | 0.916 | 0.248 | 0.395 |
| CVAI | 0.171 | 0.677 |  | 0.390 | 0.243 | 0.581 | 0.199 | 0.396 |
| TyG | 0.044* | 0.361 | 0.390 |  | 0.948 | 0.399 | 0.812 | 0.950 |
| TyG-BMI | 0.064 | 0.288 | 0.243 | 0.948 |  | 0.256 | 0.666 | 0.926 |
| TyG-WC | 0.155 | 0.916 | 0.581 | 0.399 | 0.256 |  | 0.226 | 0.438 |
| METSIR | 0.056 | 0.248 | 0.199 | 0.812 | 0.666 | 0.226 |  | 0.767 |
| AIP | 0.051 | 0.395 | 0.396 | 0.950 | 0.926 | 0.438 | 0.767 |  |
| **Female** |  |  |  |  |  |  |  |  |
| eGDR |  | 0.012* | 0.087 | 0.005* | 0.161 | 0.030* | 0.120 | <0.001* |
| LAP | 0.012* |  | 0.002* | 0.708 | 0.003* | 0.130 | 0.012* | 0.417 |
| CVAI | 0.087 | 0.002* |  | 0.111 | 0.230 | 0.016 | 0.461 | 0.013* |
| TyG | 0.005* | 0.708 | 0.111 |  | 0.004* | 0.801 | 0.016* | 0.108 |
| TyG-BMI | 0.161 | 0.003* | 0.230 | 0.004* |  | 0.014* | 0.387 | 0.001* |
| TyG-WC | 0.030* | 0.130 | 0.016* | 0.801 | 0.014* |  | 0.056 | 0.236 |
| METSIR | 0.120 | 0.012* | 0.461 | 0.016* | 0.387 | 0.056 |  | 0.001* |
| AIP | <0.001* | 0.417 | 0.013* | 0.108 | 0.001* | 0.236 | 0.001* |  |

**Abbreviation:** eGDR, estimated glucose disposal rate; LAP，Lipid Accumulation Product；CVAI, Chinese visceral adiposity index; TyG, triglyceride-glucose; TyG-BMI, TyG-body mass index; TyG-WC, TyG-Waist Circumference; METS-IR, metabolic score for insulin resistance; AIP, atherogenic index of plasma;

*p < 0.05

**Table S5.** DeLong test p-value table for insulin resistance (IR) surrogate markers and rapid decline in kidney function in men and women across different age groups.

| **name** | **eGDR** | **LAP** | **CVAI** | **TyG** | **TyG-BMI** | **TyG-WC** | **METSIR** | **AIP** |
| --- | --- | --- | --- | --- | --- | --- | --- | --- |
| **Male** |  |  |  |  |  |  |  |  |
| **45–60** |  |  |  |  |  |  |  |  |
| eGDR |  | 0.072 | 0.244 | 0.007* | 0.032* | 0.122 | 0.026* | 0.006* |
| LAP | 0.072 |  | 0.001* | 0.052 | 0.851 | 0.009* | 0.812 | 0.129 |
| CVAI | 0.244 | 0.001* |  | 0.002* | 0.047 | 0.032* | 0.042* | 0.003* |
| TyG | 0.007* | 0.052 | 0.002* |  | 0.109 | 0.006* | 0.138 | 0.776 |
| TyG-BMI | 0.032* | 0.851 | 0.047* | 0.109 |  | 0.359 | 0.864 | 0.174 |
| TyG-WC | 0.122 | 0.009* | 0.032* | 0.006* | 0.359 |  | 0.355 | 0.029* |
| METSIR | 0.026* | 0.812 | 0.042* | 0.138 | 0.864 | 0.355 |  | 0.133 |
| AIP | 0.006* | 0.129 | 0.003* | 0.776 | 0.174 | 0.029* | 0.133 |  |
| **≥60** |  |  |  |  |  |  |  |  |
| eGDR |  | 0.169 | 0.353 | 0.059 | 0.087 | 0.207 | 0.084 | 0.053 |
| LAP | 0.169 |  | 0.016* | 0.402 | 0.652 | 0.115 | 0.614 | 0.246 |
| CVAI | 0.353 | 0.016* |  | 0.089 | 0.073 | 0.046* | 0.047* | 0.035* |
| TyG | 0.059 | 0.402 | 0.089 |  | 0.653 | 0.232 | 0.696 | 0.669 |
| TyG-BMI | 0.087 | 0.652 | 0.073 | 0.653 |  | 0.390 | 0.908 | 0.491 |
| TyG-WC | 0.207 | 0.115 | 0.046* | 0.232 | 0.390 |  | 0.362 | 0.136 |
| METSIR | 0.084 | 0.614 | 0.047* | 0.696 | 0.908 | 0.362 |  | 0.434 |
| AIP | 0.053 | 0.246 | 0.035* | 0.669 | 0.491 | 0.136 | 0.434 |  |
| **Female** |  |  |  |  |  |  |  |  |
| **45–60** |  |  |  |  |  |  |  |  |
| eGDR |  | 0.011* | 0.019* | 0.006* | 0.010* | 0.015* | 0.009* | 0.005* |
| LAP | 0.011* |  | 0.541 | 0.884 | 0.824 | 0.737 | 0.911 | 0.969 |
| CVAI | 0.019* | 0.541 |  | 0.781 | 0.689 | 0.648 | 0.443 | 0.620 |
| TyG | 0.006* | 0.884 | 0.781 |  | 0.973 | 0.997 | 0.837 | 0.739 |
| TyG-BMI | 0.010* | 0.824 | 0.689 | 0.973 |  | 0.966 | 0.409 | 0.824 |
| TyG-WC | 0.015* | 0.737 | 0.648 | 0.997 | 0.966 |  | 0.760 | 0.869 |
| METSIR | 0.009* | 0.911 | 0.443 | 0.837 | 0.409 | 0.760 |  | 0.948 |
| AIP | 0.005* | 0.969 | 0.620 | 0.739 | 0.824 | 0.869 | 0.948 |  |
| **≥60** |  |  |  |  |  |  |  |  |
| eGDR |  | 0.003* | 0.012* | <0.001* | 0.009* | 0.012* | 0.002* | <0.001* |
| LAP | 0.003* |  | 0.082 | 0.412 | 0.232 | 0.052 | 0.655 | 0.080 |
| CVAI | 0.012* | 0.082 |  | 0.049 | 0.985 | 0.417 | 0.356 | 0.003* |
| TyG | <0.001* | 0.412 | 0.049 |  | 0.015* | 0.149 | 0.156 | 0.236 |
| TyG-BMI | 0.009* | 0.232 | 0.985 | 0.015* |  | 0.592 | 0.038* | 0.003* |
| TyG-WC | 0.012* | 0.052 | 0.417 | 0.149 | 0.592 |  | 0.802 | 0.032* |
| METSIR | 0.002* | 0.655 | 0.356 | 0.156 | 0.038 | 0.802 |  | 0.014* |
| AIP | <0.001* | 0.080 | 0.003* | 0.236 | 0.003* | 0.032* | 0.014* |  |

**Abbreviation:** eGDR, estimated glucose disposal rate; LAP，Lipid Accumulation Product；CVAI, Chinese visceral adiposity index; TyG, triglyceride-glucose; TyG-BMI, TyG-body mass index; TyG-WC, TyG-Waist Circumference; METS-IR, metabolic score for insulin resistance; AIP, atherogenic index of plasma;

*p < 0.05

**Table S6.**ROC curves for insulin resistance (IR) surrogate markers and the risk of CKD in men and women across different age groups.

| **Group** | **Variable** | **AUC (95% CI)** | **P for comparison** | **Optimal cutoff value** | **Sensitivity** | **Specificity** | **Accuracy** |
| --- | --- | --- | --- | --- | --- | --- | --- |
| **Male** |  |  |  |  |  |  |  |
| **45–60** | **eGDR** | 0.862(nan-nan) | 0.005* | 10.178(6.674-10.208) | 0.650(0.617-0.947) | 1.000(nan-nan) | 0.005(0.001-0.010) |
|  | **LAP** | 0.746(nan-nan) | 0.670 | 39.052(13.419-276.794) | 0.750(nan-nan) | 0.740(0.311-0.992) | 0.995(0.990-1.000) |
|  | **CVAI** | 0.696(nan-nan) | 0.510 | 120.543(59.264-216.585) | 0.750(nan-nan) | 0.734(0.247-0.993) | 0.995(0.990-1.000) |
|  | **TyG** | 0.692(0.546-0.933) | 0.386 | 8.912(8.912-9.882) | 0.750(0.387-1.000) | 0.703(0.673-0.947) | 0.994(0.989-0.999) |
|  | **TyG-BMI** | 0.661(nan-nan) | 0.144 | 257.501(210.174-290.280) | 0.500(nan-nan) | 0.909(0.613-0.979) | 0.995(0.989-0.999) |
|  | **TyG-WC** | 0.710(nan-nan) | 0.817 | 920.031(657.637-1108.328) | 0.500(nan-nan) | 0.919(0.298-0.994) | 0.995(0.990-1.000) |
|  | **METS-IR** | 0.674(nan-nan) | 0.102 | 38.703(26.077-71.470) | 0.750(nan-nan) | 0.733(0.061-0.995) | 0.996(0.990-1.000) |
|  | **AIP** | 0.725(0.396-0.944) | Reference | 0.672(0.672-1.038) | 0.750(0.371-1.000) | 0.829(0.805-0.959) | 0.862(0.804-0.997) |
| **≥60** | **eGDR** | 0.552(0.427-0.663) | 0.104 | 8.531(6.672-10.858) | 0.748(0.453-0.916) | 0.429(0.218-0.713) | 0.041(0.030-0.051) |
|  | **LAP** | 0.565(0.449-0.645) | 0.342 | 36.127(6.071-86.782) | 0.400(0.233-0.967) | 0.802(0.140-0.961) | 0.959(0.946-0.972) |
|  | **CVAI** | 0.552(0.448-0.613) | 0.309 | 154.601(42.856-172.189) | 0.229(0.143-0.935) | 0.919(0.176-0.966) | 0.957(0.945-0.969) |
|  | **TyG** | 0.608(0.504-0.720) | 0.827 | 8.926(8.098-9.006) | 0.429(0.326-0.931) | 0.802(0.253-0.845) | 0.960(0.945-0.974) |
|  | **TyG-BMI** | 0.623(0.553-0.733) | 0.608 | 214.783(177.681-246.193) | 0.486(0.256-0.821) | 0.793(0.440-0.925) | 0.961(0.948-0.970) |
|  | **TyG-WC** | 0.562(0.470-0.662) | 0.357 | 811.797(635.108-919.403) | 0.343(0.165-0.838) | 0.828(0.277-0.962) | 0.960(0.949-0.970) |
|  | **METS-IR** | 0.623(0.503-0.712) | 0.550 | 41.638(32.458-46.970) | 0.371(0.236-0.674) | 0.878(0.562-0.947) | 0.959(0.947-0.971) |
|  | **AIP** | 0.603(0.503-0.697) | Reference | 0.593(0.468-0.631) | 0.429(0.317-0.587) | 0.861(0.758-0.884) | 0.822(0.749-0.865) |
| **Female** |  |  |  |  |  |  |  |
| **45–60** | **eGDR** | 0.645(0.438-0.806) | 0.295 | 5.845(5.421-10.745) | 0.970(0.416-0.986) | 0.375(0.211-1.000) | 0.008(0.004-0.013) |
|  | **LAP** | 0.623(0.396-0.803) | 0.176 | 20.304(20.304-65.069) | 1.000(0.559-1.000) | 0.281(0.259-0.834) | 0.992(0.988-0.996) |
|  | **CVAI** | 0.565(0.357-0.758) | 0.790 | 110.060(61.233-192.226) | 0.625(0.174-1.000) | 0.603(0.135-0.986) | 0.992(0.986-0.997) |
|  | **TyG** | 0.568(0.351-0.673) | 0.332 | 8.636(8.235-9.489) | 0.750(0.500-1.000) | 0.509(0.211-0.882) | 0.993(0.988-0.997) |
|  | **TyG-BMI** | 0.575(0.327-0.796) | 0.663 | 261.779(179.759-279.158) | 0.375(0.343-1.000) | 0.887(0.219-0.949) | 0.992(0.984-0.997) |
|  | **TyG-WC** | 0.645(0.444-0.763) | 0.178 | 697.171(641.850-923.152) | 0.875(0.381-1.000) | 0.390(0.216-0.937) | 0.992(0.988-0.997) |
|  | **METS-IR** | 0.549(0.283-0.741) | 0.955 | 47.323(28.886-54.426) | 0.375(0.250-1.000) | 0.910(0.156-0.972) | 0.992(0.987-0.996) |
|  | **AIP** | 0.545(0.344-0.711) | Reference | 0.388(0.059-0.524) | 0.625(0.600-1.000) | 0.562(0.163-0.730) | 0.447(0.169-0.729) |
| **≥60** | **eGDR** | 0.580(0.504-0.664) | 0.001* | 8.766(6.550-11.061) | 0.693(0.304-0.886) | 0.487(0.286-0.927) | 0.047(0.031-0.063) |
|  | **LAP** | 0.588(0.466-0.695) | 0.155 | 40.400(28.977-97.573) | 0.590(0.190-0.824) | 0.623(0.441-0.925) | 0.955(0.937-0.967) |
|  | **CVAI** | 0.541(0.434-0.616) | 0.013* | 123.561(71.698-159.577) | 0.333(0.154-0.910) | 0.785(0.244-0.955) | 0.953(0.942-0.965) |
|  | **TyG** | 0.578(0.495-0.673) | 0.024* | 8.702(8.210-10.130) | 0.641(0.157-0.930) | 0.537(0.206-0.973) | 0.953(0.943-0.963) |
|  | **TyG-BMI** | 0.515(0.387-0.638) | 0.003* | 232.590(180.599-268.588) | 0.308(0.159-0.895) | 0.799(0.317-0.929) | 0.953(0.940-0.965) |
|  | **TyG-WC** | 0.556(0.441-0.658) | 0.054 | 790.424(724.861-975.055) | 0.487(0.104-0.799) | 0.677(0.474-0.974) | 0.953(0.937-0.964) |
|  | **METS-IR** | 0.536(0.451-0.615) | 0.003* | 42.240(24.848-47.467) | 0.282(0.183-0.995) | 0.836(0.095-0.927) | 0.955(0.942-0.964) |
|  | **AIP** | 0.645(0.567-0.706) | Reference | 0.412(0.137-0.435) | 0.667(0.573-0.987) | 0.609(0.275-0.644) | 0.552(0.307-0.646) |

**Abbreviation:** eGDR, estimated glucose disposal rate; LAP，Lipid Accumulation Product；CVAI, Chinese visceral adiposity index; TyG, triglyceride-glucose; TyG-BMI, TyG-body mass index; TyG-WC, TyG-Waist Circumference; METS-IR, metabolic score for insulin resistance; AIP, atherogenic index of plasma;

*p < 0.05

**Table S7.** DeLong test p-value table for insulin resistance (IR) surrogate markers and CKD in men and women across different age groups.

| **name** | **eGDR** | **LAP** | **CVAI** | **TyG** | **TyG-BMI** | **TyG-WC** | **METSIR** | **AIP** |
| --- | --- | --- | --- | --- | --- | --- | --- | --- |
| **Male** |  |  |  |  |  |  |  |  |
| **45–60** |  |  |  |  |  |  |  |  |
| eGDR |  | 0.001* | 0.003* | 0.005* | 0.023* | 0.004* | 0.024* | 0.005* |
| LAP | 0.001* |  | 0.049* | 0.009* | 0.053 | 0.058 | 0.243 | 0.670 |
| CVAI | 0.003* | 0.049* |  | 0.902 | 0.417 | 0.706 | 0.677 | 0.510 |
| TyG | 0.005* | 0.009* | 0.902 |  | 0.415 | 0.586 | 0.721 | 0.386 |
| TyG-BMI | 0.023* | 0.053 | 0.417 | 0.415 |  | 0.270 | 0.670 | 0.144 |
| TyG-WC | 0.004* | 0.058 | 0.706 | 0.586 | 0.270 |  | 0.599 | 0.817 |
| METSIR | 0.024* | 0.243 | 0.677 | 0.721 | 0.670 | 0.599 |  | 0.102 |
| AIP | 0.005* | 0.670 | 0.510 | 0.386 | 0.144 | 0.817 | 0.102 |  |
| **≥60** |  |  |  |  |  |  |  |  |
| eGDR |  | 0.292 | 0.340 | 0.088 | 0.087 | 0.306 | 0.085 | 0.104 |
| LAP | 0.292 |  | 0.545 | 0.272 | 0.095 | 0.760 | 0.118 | 0.342 |
| CVAI | 0.340 | 0.545 |  | 0.275 | 0.072 | 0.501 | 0.079 | 0.309 |
| TyG | 0.088 | 0.272 | 0.275 |  | 0.660 | 0.283 | 0.672 | 0.827 |
| TyG-BMI | 0.087 | 0.095 | 0.072 | 0.660 |  | 0.069 | 0.995 | 0.608 |
| TyG-WC | 0.306 | 0.760 | 0.501 | 0.283 | 0.069 |  | 0.093 | 0.357 |
| METSIR | 0.085 | 0.118 | 0.079 | 0.672 | 0.995 | 0.093 |  | 0.550 |
| AIP | 0.104 | 0.342 | 0.309 | 0.827 | 0.608 | 0.357 | 0.550 |  |
| **Female** |  |  |  |  |  |  |  |  |
| **45–60** |  |  |  |  |  |  |  |  |
| eGDR |  | 0.167 | 0.333 | 0.225 | 0.325 | 0.159 | 0.394 | 0.295 |
| LAP | 0.167 |  | 0.055 | 0.269 | 0.398 | 0.369 | 0.273 | 0.176 |
| CVAI | 0.333 | 0.055 |  | 0.961 | 0.860 | <0.001* | 0.803 | 0.790 |
| TyG | 0.225 | 0.269 | 0.961 |  | 0.927 | 0.258 | 0.792 | 0.332 |
| TyG-BMI | 0.325 | 0.398 | 0.86 | 0.927 |  | 0.154 | 0.105 | 0.663 |
| TyG-WC | 0.159 | 0.369 | <0.001* | 0.258 | 0.154 |  | 0.116 | 0.178 |
| METSIR | 0.394 | 0.273 | 0.803 | 0.792 | 0.105 | 0.116 |  | 0.955 |
| AIP | 0.295 | 0.176 | 0.79 | 0.332 | 0.663 | 0.178 | 0.955 |  |
| **≥60** |  |  |  |  |  |  |  |  |
| eGDR |  | 0.067 | 0.186 | 0.037* | 0.264 | 0.154 | 0.167 | 0.001* |
| LAP | 0.067 |  | 0.056 | 0.801 | 0.049* | 0.037* | 0.177 | 0.155 |
| CVAI | 0.186 | 0.056 |  | 0.318 | 0.400 | 0.490 | 0.882 | 0.013* |
| TyG | 0.037* | 0.801 | 0.318 |  | 0.045* | 0.563 | 0.209 | 0.024* |
| TyG-BMI | 0.264 | 0.049* | 0.400 | 0.045* |  | 0.255 | 0.196 | 0.003* |
| TyG-WC | 0.154 | 0.037* | 0.490 | 0.563 | 0.255 |  | 0.609 | 0.054 |
| METSIR | 0.167 | 0.177 | 0.882 | 0.209 | 0.196 | 0.609 |  | 0.003* |
| AIP | 0.001* | 0.155 | 0.013* | 0.024* | 0.003* | 0.054 | 0.003* |  |

**Abbreviation:** eGDR, estimated glucose disposal rate; LAP，Lipid Accumulation Product；CVAI, Chinese visceral adiposity index; TyG, triglyceride-glucose; TyG-BMI, TyG-body mass index; TyG-WC, TyG-Waist Circumference; METS-IR, metabolic score for insulin resistance; AIP, atherogenic index of plasma;

*p < 0.05
